# System dynamics of preadolescent mental wellbeing: A multi-actor perspective in Amsterdam using systems archetypes

**DOI:** 10.1177/22799036261455634

**Published:** 2026-06-24

**Authors:** Eline M. Meuleman, Vincent Busch, Wilma E. Waterlander, Nanda E. van der Poel, Carry M. Renders, Maartje M. van Stralen

**Affiliations:** 1 Department of Health Sciences and Amsterdam Public Health Research Institute, 1190 Vrije Universiteit Amsterdam, Amsterdam, Netherlands; 2 Department of Healthy Living, 650408 GGD Amsterdam, Amsterdam, Netherlands; 3 Department of Public and Occupational Health and Amsterdam Public Health Research Institute, Amsterdam UMC Location University of Amsterdam, Amsterdam, Netherlands

**Keywords:** system dynamics, systems archetypes, preadolescent, mental wellbeing, causal loop diagram

## Abstract

**Background:**

Although research into the system dynamics underlying mental wellbeing has increased over recent years, it often remains rooted in positivist approaches that overlook lived experiences and dynamic system behaviour. This paper examines the system dynamics shaping preadolescent mental wellbeing by incorporating the perspectives of diverse stakeholders and analysing the system’s overall behaviour.

**Design and methods:**

This qualitative study builds on earlier literature and scientific expert focus group data by incorporating the perspectives of preadolescents, caretakers, and professionals in Amsterdam, which were collected through a cocreated conversational tool and semistructured interviews. These insights informed a causal loop diagram (CLD), which was analysed using systems archetypes.

**Results:**

Multi-actor input from preadolescents, caretakers, and professionals expanded a science-based CLD to 62 factors, 50 identified feedback loops, and eleven subsystems. It revealed how social norms, institutional structures, and local contexts interact to shape preadolescent mental wellbeing. Seven systems archetypes were identified – mainly ‘Success to the Successful’, ‘Shifting the Burden’, and ‘Fixes that Fail’ – offering an understanding of recurring patterns that sustain (poor) mental wellbeing, from fragmented poverty support to changing societal norms around smartphone use.

**Conclusions:**

These findings underscore that complex public health issues have no quick or simple fixes. Instead, they require multilevel strategies that target various structural drivers while taking into account archetypal patterns. By mapping the system’s structure and behaviour, this study hopes to encourage policymakers, educators, and communities to adopt a systems (archetype) lens to understand preadolescent mental wellbeing and act accordingly.

**Significance for public health**

Preadolescent mental wellbeing is shaped by a wide range of interacting dynamics, from livelihood insecurity affecting caretakers’ stress and availability, to social comparison norms that pressure young people to present a ‘perfect’ image. Societal norms such as rising individualism and constant online connectivity act as powerful drivers within these dynamics. By combining system structure (via a science-based Causal Loop Diagram) with system behaviour (via system archetype analysis), this study uncovers deeper, often hidden mechanisms, including context-dependent pathways and macro-level societal and political forces. These insights highlight an urgent need for preventive, cross-sector, and equity-focused public-health strategies that address the systemic conditions shaping children’s wellbeing, rather than relying on short-term or symptom-focused solutions.

## Introduction

The mental wellbeing of preadolescents – children aged nine to twelve – has been under pressure in recent years. For example, the prevalence of emotional problems among 11- to 12-year-old girls has more than doubled, rising from 14% to 33% between 2017 and 2021.^1^ These patterns are particularly concerning given that many adult mental disorders originate during (early) adolescence, a critical neurodevelopmental period. This underscores the need for early action strategies to support mental wellbeing.^2,3^ To inform the design and prioritization of such action strategies, insight into the factors that shape preadolescent mental wellbeing is needed.^4^

Previous research has focused on isolated factors affecting preadolescents’ mental wellbeing, such as the quality of friendships or the impact of poverty.^5–7^ While these studies identified relevant associations, no single factor can fully explain the prevalence of preadolescent mental wellbeing.^8^ Indeed, the interdependence between individuals and their environment has long been emphasized in psychological theory. For example, Bandura’s Social Cognitive Theory conceptualizes this through reciprocal determinism, in which personal factors, behaviour, and environmental context continuously interact.^9,10^ Similarly, Bronfenbrenner’s ecological systems theory posits that child development is shaped by interconnected environmental systems, ranging from the immediate microsystem (family) to the broader macrosystem (societal and cultural contexts).^7,11^ However, system dynamics theory takes this a step further, by examining the emerging dynamic interactions over time.^12^

The use of approaches based on system dynamics theory to understand mental health has increased, primarily in clinical samples. Applications range from suicidal behaviour^13^ to treatment access for individuals with eating disorders^14^ and depression.^15^ In a recent study,^16^ we explored the system dynamics underlying preadolescent mental wellbeing from a scientific perspective on the basis of an umbrella review and focus groups with scientists. This study revealed the interplay between the individual, familial and societal levels. The identified dynamics revolved around, for instance, societal pressure to perform, identity formation, parenting stress, the norm to be always online, and neighbourhood constraints, each embedded within broader systemic forces beyond individual control.

Much of the expanding literature on preadolescent mental wellbeing has adopted positivist, measurement-driven designs, whereas interpretive approaches that capture stakeholders’ lived experiences and contextual mechanisms are underrepresented. Indeed, gathering the perspectives of relevant actors is deemed crucial to better understand how the system operates and how local social, political, and cultural dynamics shape mental wellbeing.^17–19^ As illustrated by recent research on preadolescents’ online experiences, such qualitative methods can uncover nuanced, lived perspectives, such as context-dependent online challenges that rapidly change and are often overlooked in quantitative studies.^20^

Moreover, in current research, system-based analyses have focused on identifying factors, feedback loops, and subsystems, such as the *structure* of a system. However, ultimately, it is also of interest to understand the *behaviour* of the system, which refers to how the system acts over time and which dynamic patterns (“systems archetypes”) emerge from the system structure.^21^ The aim of this paper is therefore to gain a richer understanding of the system dynamics driving preadolescent mental wellbeing by (a) including the perspectives of diverse stakeholders and (b) analysing the behaviour of the system as a whole.

## Methods

### Study design

This qualitative study expanded upon previously collected data from the scientific literature and focus groups with scientific experts^16^ with the perspectives of preadolescents, caretakers and professionals living (or working) in Amsterdam. Perspectives of preadolescents and their caretakers were gathered through a cocreated conversational tool, whereas professionals’ perspectives were collected via individual semistructured interviews. These perspectives informed the development of a causal loop diagram (CLD), which was subsequently analysed via systems archetypes to identify the underlying system dynamics.

Ethical approval for this study was granted by the VU University Medical Ethical Committee (study protocol 2023.0781). Professionals who were interviewed received an information letter and provided written informed consent before participation. No personal or sensitive data were collected from caretakers or preadolescents; therefore, no informed consent was required from them.

### Study setting

This study focused on three neighbourhoods within Amsterdam that prioritized youth mental health within their local policy agendas. Beginning in early 2024, these neighbourhoods committed to a six-year trajectory aimed at progressing from systems understanding to systems change. The present study represents the initial phase of this process. The neighbourhoods – Indische Buurt, Gaasperdam, and Oud-Noord – are characterized by relatively high proportions of residents with lower socioeconomic positions, with populations of 42,611, 32,593, and 32,250, respectively, in 2024.

### Data collection and participants

#### Preadolescents: Conversations using “the tree”

**Figure 1.**
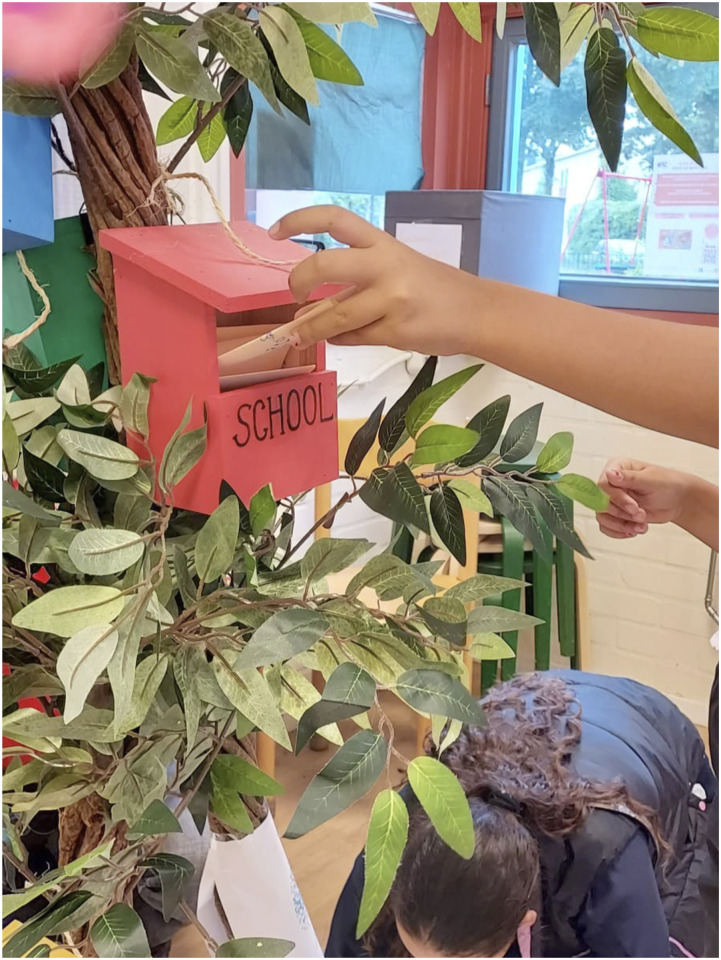
A picture made during data collection with *The Tree*.

To explore how preadolescents experience and interpret their mental wellbeing, we cocreated a conversational method called *The Tree* together with youth professionals from a local youth organization (Figure 1). One youth professional took the lead in creating the idea and prototype, and one interactive meeting was held to gather the ideas of six other professionals. All professionals were embedded in the local context, which ensured that this method was easy for both local preadolescents and professionals to use. *The Tree* is a 180 cm artificial tree featuring four brightly coloured birdhouses representing four key environments: home, school, online, and leisure. For each birdhouse, there was a ‘wellbeing card’ in the same colour as the birdhouse, containing an emotion thermometer and prompt. Preadolescents could fill in this card together with a trusted professional, and place it in the corresponding birdhouse. The trusted professionals (such as youth and child workers who are already familiar with the children) used predetermined prompts to spark brief, playful conversations that focus on the underlying causes of their state of mental wellbeing, as depicted on the card in that specific environment.

This concept draws on the tree metaphor previously used in system dynamics literature, which illustrates the interconnectedness between different elements: leaves represent the thoughts and behaviours of preadolescents; branches symbolize the underlying patterns and trends that support these thoughts and behaviours; the trunk represents the structures and drivers; and the roots represent the deeper social paradigms.^22^ *The Tree* was designed as a child-focused, age-appropriate conversational tool. Prior research on interviewing young children shows that visual or projective techniques can help create a low-threshold setting, comfort children by doing something while talking, and thereby support children in sharing their experiences more freely than direct questioning alone.^23^

We implemented *The Tree* in partnership with at least one local organization per neighbourhood – including youth work groups, playground-based child services, teen coaching services, and a chess school – each using the method for at least three weeks during both regular and special activities. The professionals used convenience sampling to include a broad range of preadolescents in the study. All children aged 9-12 years were eligible to participate anonymously, as long as they lived within one of the three participating neighbourhoods. Preadolescents filled in between one and four wellbeing cards. The professionals were encouraged to collect cards across all four birdhouses to ensure that underlying causes were captured for each environment. Before the implementation, professionals received one hour of training on the method. Afterwards, professionals were invited for a 1.5-hour semistructured interview with the research team (EM or NvdP), during which they were asked to share insights from the conversations. The interviewers asked follow-up questions to help distinguish between what professionals heard from preadolescents and their own interpretations.

#### Caretakers: Conversations using “the tree”

*The Tree* was also used to explore caretakers’ perspectives. Researchers (EM, NvdP) distributed wellbeing cards among caretakers based on convenience sampling, again featuring an emotion thermometer and prompts for each key environment (home, school, online, leisure) to spark conversations about what influences their child’s mental wellbeing. These researcher-led conversations took place in familiar community settings such as school playgrounds and neighbourhood events. Inclusion criteria for caretakers were that they took care for at least one child between nine and twelve years old and lived within one of the three participating neighbourhoods. The conversations lasted approximately 15-30 minutes, were anonymous and not audio-recorded. Researchers documented key insights in short summaries immediately after the conversations. For these conversations with caretakers, data collection continued until no substantially new underlying causes of mental wellbeing across the four environments came to light.^24^

#### Professionals: Interviews

Professionals working with preadolescents (e.g., in education, health care, or sports) were invited via email for one-hour, semistructured interviews conducted either in person or online (with EM or NvdP). Interviews were audio-recorded. Invitations were distributed through existing professional networks of collaborating colleagues working within the municipality, and snowball sampling was used to further include professionals. Professionals were eligible if they worked with children aged 9-12 years in one of the three participating neighbourhoods. The interviews followed a structured protocol for interview-based methods for mapping mental models^25^ and explored professionals’ perspectives on the factors influencing preadolescents’ mental wellbeing. Guiding questions focused on potential feedback loops and perceived underlying mechanisms, using follow-up probes (e.g., “Why is that?”, “What is the consequence…?”) to explore underlying dynamics. All the interviews concluded with the following question: “How is it that, despite all efforts, the mental wellbeing of preadolescents remains relatively low in this neighbourhood?” Data collection continued until thematic saturation was reached within each neighbourhood, meaning that no new issues or insights emerged.^24^

### Data analysis

#### Qualitative analysis

Data from preadolescents and caretakers were transcribed and coded in MS Excel. The interview data of professionals were transcribed and coded in MAXQDA 2018. First, parts of the interview (i.e., one or multiple sentences) in which professionals spoke about factors or relations impacting preadolescent mental wellbeing were highlighted in MAXQDA. These were also given an associated code corresponding to the causal structure (factor/link/loop). Then, quotation comments were made that reflected the causal structures as described in the highlighted text in more detail (e.g., factor A → + factor B → - factor C). To do so, a codebook including causal structures, which is based on the previously developed science-based CLD,^16^ was used to assess whether the factors, links or loops mentioned in the data were already represented. If so, the same terminology was applied (deductive coding). If a new factor, link, or loop was identified, it was inductively added to the codebook and marked as a novel element. One researcher (EM) conducted the initial coding, which was reviewed by a second researcher (NvdP). Disagreements were discussed within the broader research team.

The coding process resulted in a query report per neighbourhood containing quotations, associated codes, and quotation comments containing causal structures in causal-loop diagram notations. This report was used for a brief thematic analysis of the novel elements, guiding the identification of mechanisms (see “System-based analysis: Mechanisms and archetypes”).

#### Development of a causal loop diagram

The CLD functions as a meta-level framework that synthesizes diverse individual trajectories into a model and thus does not apply uniformly for all preadolescents or their families. A CLD visualizes interconnected factors linked by positive or negative causal relationships. Some relationships involve a time gap between a change in one element and its impact on another. Together, some relationships form reinforcing and/or balancing feedback loops. In the reinforcing loops, changing the value of a variable within the loop in either direction is amplified by the action of the loop. In contrast, in a balancing loop, changing the value of a variable in either direction is counteracted or offset by the action in the loop. This is shown via a ‘delay’ symbol, represented by two short parallel lines. Altogether, the loops help to uncover the underlying mechanisms that shape system behaviour over time.

A first draft CLD was created for the first neighbourhood by expanding the existing science-based CLD^16^ with newly identified causal structures (factors, connections, and loops). The mapping followed an iterative process across the three neighbourhoods, whereby each version of the CLD was built on and the previous one was refined. In the final version, duplicate connections representing both direct and indirect connections were removed to improve readability and usability. The CLD was supplemented with a table containing the identified feedback loops. The CLD comprises numerous interconnected feedback loops; the loops identified represent the most salient loops identified by the researchers during the mapping process. The CLD and table were subsequently validated by the research team, which comprised six scientific experts. The process resulted in the creation of a multi-actor CLD map of the system of mental wellbeing in preadolescents, reflecting the system’s *structure*.

#### System-based analysis: Mechanisms and archetypes

Finally, a systems archetype analysis was conducted to gain insight into the system’s behaviour. Systems archetypes represent common, recurring patterns of system behaviour, and they can be used as tools to interpret embedded system dynamics^21,26^. Eight archetypes are particularly well known and frequently applied^21^ (Table 1).

**Table 1.** Systems archetypes and explanations.

Archetype	Description
Fixes that fail	A quick solution is applied to relieve symptoms, but it generates delayed, unintended consequences that exacerbate the original problem.
Drifting goals	A gap between a goal and current reality leads to a gradual lowering of goals, resulting in declining system standards over time.
Growth and underinvestment	Growth slows as capacity constraints emerge, but reluctance to invest reinforces the stagnation and validates the initial underinvestment.
Shifting the burden	A symptomatic solution diverts attention from a fundamental solution, eventually weakening the system's ability to address root causes.
Success to the successful	Competing groups draw from shared resources, with initial success enabling greater access and widening inequality.
Tragedy of the commons	Shared resources are overused by individual actors, leading to depletion that harms all users in the system.
Limits to success	Initial efforts yield growth until they encounter systemic constraints that dampen further progress regardless of continued efforts.
Escalation	Parties engage in actions that are perceived as threats by one another, each trying to outdo the other in a self-reinforcing cycle.

As a first step in the system analysis, mechanisms were described. The mechanisms were not only based on the system’s structure as captured in the CLD but also on the query reports of the data derived from the conversations via *The Tree* and the interviews. These mechanisms typically consisted of a combination of reinforcing and balancing feedback loops and provided a qualitative description of the system behaviour. In a second step, the mechanisms were used to iteratively identify characteristic system behaviours explaining or underlying these mechanisms that aligned with the systems archetypes listed in Table 1. Once a potential archetype was identified, we first agreed upon its storyline and then developed the archetype CLDs. One researcher (EM) first identified the archetypes, which were reviewed by the other researchers of the team in an iterative process of multiple rounds. Where interpretations remained uncertain, we verified them with interviewed professionals or consulted external professionals. This review process was used to determine the appropriateness of the mechanisms to the specific archetypes and the terms used within the archetypes, resulting in a table containing mechanisms and archetypes.

#### Research team and qualitative rigor

The study team consisted of a PhD student in preadolescent mental wellbeing from a systems perspective, with a background in public administration and psychology (EM), a researcher with a background in sociology (NvdP), a professor with expertise in integrated community-based approaches to health promotion (CR), an associate professor specialised in system dynamics approaches (WW), an associate professor in mental wellbeing (MvS), and a researcher in the public health field with expertise in health behaviour and systems (VB). The combined experience of this multidisciplinary research team enabled us to triangulate our specific areas of expertise, discuss and identify potential blind spots and thereby reduce the likelihood that important dynamics would be missed. EM and NvdP conducted the interviews. Both were female researchers affiliated with the university, had completed training in qualitative research methods, and had prior experience conducting interviews. Interviewees were not known to the interviewers prior to the study, and the interviewers did not share any personal goals or opinions during the interviews.

Given the interpretative nature of the methods and the embedded role of the researchers in the collection and analysis of the data, we used a combination of triangulation, reflexive discussions and iterative analysis to enhance qualitative rigor. Multiple data sources were integrated to inform the CLD, including scientific evidence and the perspectives of preadolescents, caretakers and professionals from three different neighbourhoods. The interviews followed a structured protocol to improve rigor.^25^ Cross-neighbourhood comparison of results supported analytical consistency while preserving contextual nuance. Coding, CLD development, and archetype identification were conducted iteratively within the multidisciplinary research team, going back and forth between the collected data and the CLD, allowing interpretations and underlying dynamics to be continuously discussed, challenged, and refined. In addition, targeted member checking with professionals for the archetype analysis was conducted.

## Results

### Participants

In total, 233 *wellbeing cards* from preadolescents and 83 *wellbeing cards* from caretakers were collected. The exact number of participating preadolescents was unclear, as each individual could submit up to four cards- one for each environment -if they chose to do so. Additionally, interviews were conducted with two groups of professionals: eleven professionals who acted as spokespersons for preadolescents they had spoken to using *The Tree* and 45 professionals who were interviewed on the basis of their own professional experiences. The data were evenly distributed across the three neighbourhoods.

### Results from the conversations and interviews

All the factors in the science-based codebook were mentioned by the participants during the conversations and interviews. Multi-actor data collection, however, also revealed several new themes that were not included in the original science-based codebook. These themes included caretakers’ ambitions leading to greater performance pressure, digital influences and their effects on the desire for wealth and success, challenges around setting rules for screen use and caretakers’ digital literacy, lack of emotional rest at home due to housing issues, adultification and exposure to risky environments, cultural norms around privacy and help-seeking, the role of (positive) role models in identity formation, overprotective caretakers and (social norms regarding) outdoor play, caretakers’ distrust in institutions and barriers to the uptake of social services and care, discrimination and social exclusion, language barriers and social cohesion, and school programmes for wellbeing.

### System structure: Causal loop diagram

**Figure 2.**
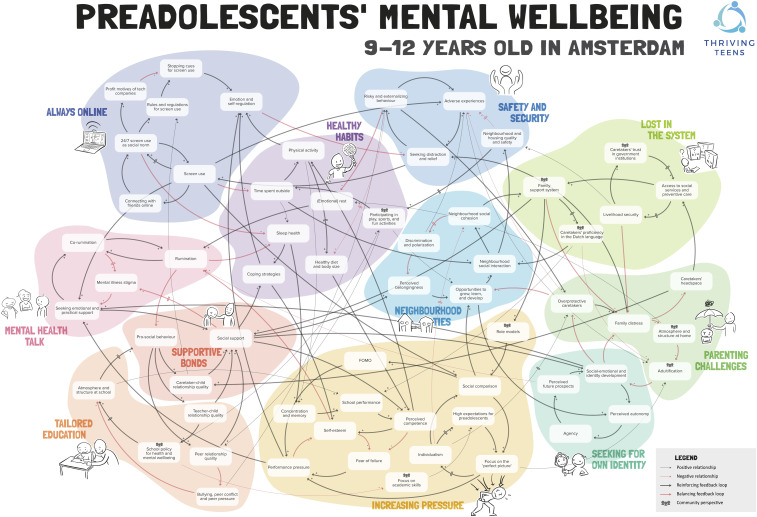
The multi-actor CLD representing the system dynamics influencing preadolescents’ mental wellbeing.

Following the integration of the multi-actor data with the science-based CLD, the final multi-actor CLD (Figure 2) consists of 62 factors, 50 identified feedback loops and eleven subsystems. Table 2 shows the identified feedback loops within each subsystem along with the overarching mechanisms that were identified through them. Mechanisms – or parts of mechanisms – based on the conversations and interviews were added in blue. Figure 2 displays only those connections that form part of a feedback loop or mechanism listed in Table 2; other connections were omitted to reduce visual complexity. See the supplementary files for figures per subsystem, which were produced to increase readability.

**Table 2.** System dynamics within the multi-actor CLD: subsystems, feedback loops, and mechanisms.

Name of subsystem	Loop nr.	Main feedback loops within the subsystem	Mechanisms
Increasing pressure	R1/R2	↑ Individualism	The societal norm of emphasizing individual achievement and performance imposes high expectations for preadolescents by themselves, peers, parents, and/or schools. **These high expectations from parents primarily stem from their desire for their children to have a better life than they themselves had, motivating them to focus on academic skills. **Simultaneously, this focus on individual achievement and performance encourages preadolescents to compare themselves to others (e.g., noting physical appearances that shape their body image, or comparing friendships and social circles that influence their sense of popularity). As a result of these high expectations and the social comparisons, preadolescents often feel the need to focus on reaching ‘a perfect picture’; to excel in academics, extracurricular activities, and personal appearance. **Preadolescents as young as eight or nine years old go to homework class after school.** This pursuit of perfection supports and reinforces the individualistic societal norm.
		↑ High expectations for preadolescents OR	
		↑ Social comparison	
		↑ Focus on the ‘perfect picture’	
		↑ Individualism	
	R3/R4	↑ Screen use	​
		↑ Social comparison	Screen use is one of the triggers of social comparisons. Social media can trigger feelings such as FOMO (fear of missing out) and the pressure to achieve perfection. The problematic situation arises when these comparisons focus on idealized online versions of peers and influencers, which then often leads to increased performance pressure. For example, many preadolescents mentioned **wanting to become rich, inspired by role models such as influencers and vloggers who seem to have achieved wealth easily. Seeing people from similar backgrounds succeed online makes this goal feel attainable. **This pressure can cause preadolescents to feel emotionally overwhelmed, less able to self-regulate their behaviour, and in turn they could seek distraction and relief through further screen use, a coping mechanism. Screen use might bring them comfort, entertainment, or social connection. This could mean endlessly scrolling through feeds, watching videos, or engaging in gaming – all of which provide a temporary escape from the stress. However, this increased screen time exposes them once again to curated images of success, beauty, or happiness.
		↑ FOMO OR ↑ Focus on reaching the ‘perfect picture’	
		↑ Performance pressure	
		↓ Emotion and self-regulation	
		↑ Seeking distraction and relief	
			**School performance may be hampered by several factors, such as the absence of a supportive and structured home environment (for instance when caretakers provide little encouragement or when the household is too busy and distracting for preadolescents to focus on homework). In addition, when caretakers have limited proficiency in Dutch, they may struggle to support their children with school-related tasks, communication with teachers, or understanding school requirements, which can make it more difficult for children to keep up in a Dutch school.** Preadolescents who perform less well at school can have a reduced perceived competence, also lowering their self-esteem. Reduced self-esteem increases rumination, which undermines sleep health. Poor sleep then impairs concentration and memory, further lowering school performance, thereby reinforcing the spiral. This loop is closely linked to performance pressure, which is influenced by fear of failure, a focus on the ‘perfect picture’ and FOMO. Higher performance pressure diminishes self-esteem, which heightens fear of failure. Increased fear of failure feeds back into greater performance pressure.
			For preadolescents facing performance pressure, actively seeking and receiving emotional or practical support (e.g. mindfulness, a shoulder to cry on) can help to develop healthy coping strategies and a higher perceived competence. The strategies and competence, in turn, improve self-esteem. With higher self-esteem, young teenagers may experience less fear of failure, which helps decrease the pressure to perform.
		↑ Screen use	
	R5	↑ Performance pressure	
		↓ Self-esteem	
		↑ Fear of failure	
		↑ Performance pressure	
	B6/B7	↑ Performance pressure	
		↑ Seeking emotional and practical support	
		↑ Social support	
		↑ Coping strategies OR ↑ Perceived competence	
		↑ Self-esteem	
		↓ Fear of failure	
		↓ Performance pressure	
	R8	↓ School performance	
		↓ Perceived competence	
		↓ Self-esteem	
		↓ (Emotional) rest	
		↑ Rumination	
		↓ Sleep health	
		↓ Concentration and memory	
		↓ School performance	
Seeking for own identity	R1/R2	↑ Agency	Preadolescence is often a time when children begin to form their own sense of identity. This identity development can be positively impacted through more opportunities to grow, learn, and develop, but hindered by overprotective parenting. When preadolescents feel a greater sense of agency over their lives, it can enhance their perception of prospects because they see themselves as capable of influencing the world around them. Perceiving good future prospects leads to preadolescents ruminating less and contributes positively to their social-emotional identity development, fostering a stronger sense of autonomy. In turn, this autonomy reinforces their sense of agency.
		↑ Perceived future prospects	
		↑ Social-emotional and identity development	
		(↓ Overprotective caretakers	​
			**Preadolescents may become adultified due to various causes, such as growing up in large families with caregiving duties, exposure to adverse or risky behaviours (e.g., drug-related crime), pressure to maintain a “perfect picture” through appearance and social image, or caretakers struggling with the Dutch language. This premature pressure to behave like an adult reduces the space and freedom preadolescents need to have a healthy social-emotional and identity development. As a result, they may struggle with challenges related to their development, placing additional strain on family relationships. As family distress increases, the atmosphere and structure at home may become more tense or less supportive, prompting preadolescents to take on even more adult-like responsibilities. This reinforces the cycle of adultification. At the same time, a balancing mechanism may also occur: when adultification undermines preadolescents’ social-emotional and identity development, caretakers may respond by becoming more overprotective. This overprotection reduces preadolescents’ exposure to adverse experiences, which in turn lowers the pressure for them to take on adult-like responsibilities. In this way, overprotective caretakers can counteract the cycle of adultification.**
		↑ Opportunities to grow, learn, develop)	
		↑ Perceived autonomy	
		↑ Agency	
	R3	↑ Adultification	
		↓ Social-emotional and identity development	
		↑ Family distress	
		↓ Atmosphere and structure at home	
		↑ Adultification	​
			**When preadolescents have access to inspiring (positive) role models in their environment, such as older youth, engaged adults, or teachers, they gain constructive examples to follow. Positive role models can help teenagers better understand themselves through social comparison, prompting them to ask: Who do I want to be? What suits me? This motivates them to seek new opportunities for growth, learning, and development, such as playing outside, joining sports clubs or engaging in creative activities. These interactions strengthen social cohesion in the neighbourhood, fostering a safe and supportive community. In a close-knit neighbourhood, families receive more support, providing a stable foundation for teenagers to further explore their identity. Beyond offering new role models, such an environment also enables teenagers to become role models themselves for younger children or their peers.**
	B4	↑ Adultification	
		↓ Social-emotional and identity development	
		↑ Overprotective caretakers	
		↓ Adverse experiences	
		↓ Adultification	
	R5	↑ Role models	
		↑ Social comparison	
		↑ Opportunities to grow, learn, develop	
		↑ Neighbourhood social interaction	
		↑ Neighbourhood social cohesion	
		↑ Family support system	
		↑ Role models	
Parenting challenges	R1	↑ Overprotective caretakers	Caretakers can be overprotective, partly because an individualistic society makes them feel like they must protect their children and help them be successful. This overprotectiveness may limit the adverse experiences preadolescents encounter (because they often have more rules) but at the same time, the feeling of needing to be very protective often leads caretakers to experience distress, because they cannot control everything. Family distress makes caretakers (emotionally) less available, which may cause children to seek for distraction and relief. One – albeit negative – way of seeking distraction is through engaging in risky and externalizing behaviour, which will intensify their parents’ perceived needs to protect them.
		↑ Family distress	
		↑ Seeking distraction and relief	
		↑ Risky and externalizing behaviour	
		↑ Overprotective caretakers	
	R2	↑ Family distress	The mental wellbeing of caretakers significantly influences that of their children, both directly and indirectly. When caretakers experience distress,** it directly impacts the atmosphere and structure at home**. Distress can also limit caretakers’ capacity to focus on parenting effectively, which can negatively impact their relationship with their child. As a result, children may receive less social support, which can hinder their identity development. When their children’s identity development is hampered, it often affects caretakers as well, because caretakers are emotionally invested in their children’s wellbeing.
		↓ Caretakers’ headspace	
		↓ Caretaker-child relationship quality	
		↓ Social support	
		↓ Social-emotional and identity development	
		↑ Family distress	One major source of distress for caretakers is financial insecurity. Living in financial insecurity often leads to preadolescents not being able to get all opportunities to grow, learn, and develop. The distress itself can reduce caretakers’ headspace. This lack of headspace can hinder caretakers from accessing appropriate social services, such as municipal aid for poverty alleviation, since they have less time and energy. This perpetuates their financial insecurity and consequently their lack of headspace for parenting. **An important buffer for caretakers’ stress – as well as for preadolescents themselves – is their own (family) support system, including friends, neighbours, community- and family members.**
	R3	↓ Livelihood security	
		↑ Family distress	
		↓ Caretakers’ headspace	
		↓ Access to social services	
		↓ Livelihood security	
Neighbourhood ties	R1/R2	↓ Neighbourhood social cohesion	Neighbourhoods with low social cohesion tend to experience higher levels of discrimination and lower levels of social support. When social cohesion and the sense of belonging are low, for instance because of high individualism in society, preadolescents face fewer opportunities to grow, learn, and develop due to limited social support and reduced neighbourhood safety (e.g., children spend less time playing outdoors because this is not safe). **Discrimination and polarization contribute to feelings of isolation among preadolescents and their caretakers, especially for those who look or speak differently from the majority.** This lack of cohesion thus often leads to a decline in neighbourhood social interactions, further weakening the community's overall sense of connectedness.
		↑ Discrimination OR ↑ Family support system AND ↑ Social support	
		↓ Perceived belongingness	
		↓ Opportunities to grow, learn, develop	​
			Livelihood security plays a role in shaping these dynamics. Families with limited livelihood security are more likely to reside in neighbourhoods and houses with poorer quality and lower safety. These conditions not only restrict preadolescents’ opportunities to grow, learn and develop (because e.g., it is unsafe to play outside unsupervised) but also reduce opportunities for positive neighbourhood interactions, as residents may feel unsafe or disconnected from one another.
		↓ Neighbourhood social interaction	
		↓ Neighbourhood social cohesion	
	R3	↑ Participating in play, sports, and fun activities	​
			**Preadolescents who participate in play, sports, and fun activities – such as after-school sports programs or playing in local playgrounds – spend more time outdoors (and less time indoors). This promotes social interaction among preadolescents in the neighbourhood; they get to know each other better and can build new friendships. As a result, the overall quality and safety of the neighbourhood improve. After all, when more preadolescents use a playground, for example, it encourages others to join in and play outside as well. In other words, when participation in play, sports, and fun activities becomes the norm, it motivates others to take part. A crucial condition is that sufficient and accessible sports and play facilities are available, which is more often the case in neighbourhoods with a higher socioeconomic status.**
		↑ Time spent outside	
		↑ Neighbourhood social interaction	
		↑ Neighbourhood and housing quality and safety	
		↑ Participating in play, sports, and fun activities	
Safety and security	R1	↑ Adverse experiences	Preadolescents can experience adverse situations from various sources such as unsafe neighbourhoods, **criminal activity,** bullying and peer conflict, or discrimination. These adverse experiences can trigger preadolescents to seek for distraction and relief. In response, preadolescents may seek distraction and relief through risky and externalizing behaviours, such as rude and boundary-breaking behaviour **or unhealthy eating behaviour (e.g., eating a lot of fast food)**. This increases the likelihood that they encounter more adverse experiences. A family’s general livelihood security impacts in turn both the quality and safety of the neighbourhood and house, and increases chances for risky and externalizing behaviour such as criminal behaviour.
		↑ Seeking distraction and relief	In the online context, this cycle can manifest as increased screen use. More time spent online heightens the chances of encountering adverse experiences, such as exposure to harmful content or cyberbullying. These experiences may then drive preadolescents to further seek distraction and relief through additional screen use, turning to their screens for comfort, entertainment, or social connection.
		↑ Risky and externalizing behaviour	
		↑ Adverse experiences	
	R2	↑ Screen use	
		↑ Adverse experiences	
		↑ Seeking distraction and relief	
		↑ Screen use	
Healthy habits	R1	↑ Screen use as social norm	Healthy habits interact with and impact the daily lives of preadolescents. One example is the common practice of using screens in the evening, which can negatively affect one’s sleep quality and duration. Poor sleep health, negatively affected by the described late night and/or nightly smartphone use, influences preadolescents’ coping with emotion and self-regulation, making them more likely to seek distraction and relief in their screens, fuelling screen use as a social norm.
		↓ Sleep health	
		↓ Coping strategies	
		↓ Emotion and self-regulation	Additionally, screen use competes with and takes away time spent outdoors – especially when outdoor play facilities are scarce – and thus decreasing physical activity. This reduced physical activity then negatively affects preadolescents’ emotion and self-regulation capabilities, leaving them vulnerable to the seductive pull of their smartphone and other screens and further increasing their screen time.
		↑ Seeking distraction and relief	
		↑ Screen use	​
			Once active, preadolescents are more likely to increase their subsequent activity. That is, higher levels of physical activity support a healthier diet and body size, which enhances preadolescents’ perceived competence. This improved sense of competence can further motivate them to engage in physical activity. Moreover, increased physical activity also boosts concentration and memory, and therefore school performance.
		↑ 24/7 screen use as social norm	
	R2	↑ Screen use	
		↓ Time spent outside	Another loop within the ‘healthy habits’ subsystem involves the role of emotional rest. **(Emotional) rest of preadolescents is largely influenced by the atmosphere and structure at home. For instance, whether they have their own bedroom or have to share it with siblings.** A lack of emotional rest can lead to increased (pre-sleep) rumination which disrupts sleep quality. Sleep health is crucial for emotional and self-regulation (e.g., reduced impulse control). To cope with these challenging emotions, preadolescents may turn to distractions and seek relief through risky or externalizing behaviours - such as excessive social media or video gaming, experimenting with alcohol, or engaging in vandalism; this, ironically, further erodes their emotional rest, adding fuel to the fire of this cycle. **Alternatives such as compelling after-school activities or engaging playgrounds can temper this negative cycle of seeking for distraction and relief.**
		↓ Physical activity	
		↓ Emotion and self-regulation	
		↑ Seeking distraction and relief	
		↑ Screen use	
	R3	↑ Physical activity	
		↑ Healthy diet and body size	
		↑ Perceived competence	
		↑ Physical activity	
	R4	↑ Physical activity	
		↑ Concentration and memory	
		↑ School performance	
		↑ Perceived competence	
		↑ Physical activity	
	R5	↓ (Emotional) rest	
		↑ Rumination	
		↓ Sleep health	
		↓ Coping strategies	
		↓ Emotion and self-regulation	
		↑ Seeking distraction and relief	
		↑ Risky and externalizing behaviour	
		↓ (Emotional) rest	
Always online	R1	↑ Profit motives of tech companies	Capturing people's attention as much and for as long as possible is the main money maker and driving force that shapes the design of (social media) apps and digital space in general. Designs that lack stopping cues are aimed at seducing its users, making emotion- and self-regulation difficult leading to users seeking distraction and relief in using their screens. The social norm is shifted towards **24/7 screen use as a social norm**, making (a lot of) screen use more socially accepted and even expected. **Preadolescents can’ keep up with the newest trends and news, when not checking their smartphone. The new social norm** increases profits for tech companies, who then have more incentive to design even more addictive features.
		↓ Stopping cues for screen use	
		↓ Emotion and self-regulation	
		↑ Seeking distraction and relief	
		↑ Screen use	Rules and regulations that limit screen time directly affect preadolescents’ screen use. These rules can come from various sources, such as parents setting daily screen time limits or prohibiting phone use in the bedroom, schools banning screens during class hours, or national laws restricting social media use for certain age groups. Stricter rules on screen use help introduce (external) stopping cues, which support the development of emotional control and self-regulation skills, preventing users from seeking distraction and relief through screens. This leads to a reduction in screen time, weakening its status as a social norm. As screen use declines, the need for strict regulations decreases as well.
			Simultaneously, screen use has a positive side in that it allows one to connect with friends and seek and receive emotional and social support in online communities. Preadolescents often find it easier to disclose their feelings behind the safety of a screen. By receiving this support and developing healthy coping strategies and subsequently better emotion- and self-regulation, preadolescents may reduce their (passive, e.g., scrolling through social media, playing games) screen time in the end. At the same time, screen use can amplify itself through two reinforcing channels: (1) spending time online with friends strengthens peer bonds and makes screen-based interaction feel normative, increasing screen use; and (2) even without direct peer contact, mere exposure to widespread screen use in families, media, and daily routines normalizes screen use as a social norm, which independently reinforces additional screen time.
		↑ 24/7 screen use as social norm	
		↑ Profit motives of tech companies	
	B2	↑ Rules and regulations for screen use	
		↑ Stopping cues for screen use	
		↑ Emotion and self-regulation	
		↓ Seeking distraction and relief	
		↓ Screen use	
		↓ 24/7 screen use as social norm	
		↓ Rules and regulations for screen use	
	B3	↑ Screen use	
		↑ Seeking emotional and practical support	
		↑ Social support	
		↑ Coping strategies	
		↑ Emotion and self-regulation	
		↓ Seeking distraction and relief	
		↓ Screen use	
	R4	↑ Screen use	
		↑ Connecting with friends online	
		↑ 24/7 screen use as social norm	
		↑ Screen use	
	R5	↑ Screen use	
		↑ 24/7 screen use as social norm	
		↑ Screen use	
Mental health talk	R1	↓ Mental illness stigma	The growing normalization of discussing mental illnesses alongside the declining mental illness stigma has positive effects, such as encouraging preadolescents to seek (online) emotional and practical support from professionals. This leads to increased perceived social support and reduces the stigma around talking about for instance perceived future prospects and family distress.
		↑ Seeking emotional and practical support	
		↑ Social support	​
			However, when preadolescents seek support from each other, they (especially girls) may also engage in co-rumination. Co-rumination is the process of extensively discussing and rehashing problems, negative feelings, and concerns. This focus on the negative can increase rumination and further increase co-rumination, thereby normalizing mental illnesses and reducing mental illness stigma – but in a negative way. For example, preadolescents who co-ruminate and ruminate more often can interpret normal feelings like occasional sadness as signs of depression.
		↓ Mental illness stigma	
	R2	↓ Mental illness stigma	
		↑ Seeking emotional and practical support	
		↑ Co-rumination	
		↑ Rumination	
		↑ Co-rumination	
		↓ Mental illness stigma	
Supportive bonds	R1 – R6	↑ Social support	Social support can help preadolescents break free from the cycle of rumination. Preadolescents who experience social support, tend to develop stronger coping strategies and a greater sense of perceived competence, both fostering self-esteem. When preadolescents ruminate less, they are more prone to engage in pro-social behaviour, fostering stronger relationships with peers (also online), teachers, and caretakers. These improved relationships, in turn, enhance both the received and perceived social support, buffering them against distress. In addition, preadolescents – who have a high social-emotional and identity development – are more likely to seek emotional and practical support from their significant others, increasing their social support.
		↑ Coping strategies OR ↑ Perceived competence	
		↑ Self-esteem	
		↓ Rumination	
		↑ Pro-social behaviour	
		↑ Peer relationship quality OR ↑ Teacher-child relationship quality OR ↑ Caretaker-child relationship quality	
		↑ Social support	
	R7	↑ Social-emotional and identity development	
		↑ Seeking emotional and practical support	
		↑ Social support	
		↑ Social-emotional and identity development	
Lost in the system	R1	↓ Livelihood security	One major source of distress for caretakers is financial insecurity, reducing their headspace. **In Amsterdam, a high number of caretakers are either unemployed or single parents, which increases financial insecurity. **This lack of headspace can hinder caretakers from accessing appropriate social services, such as municipal aid for poverty alleviation, since they have less time and energy. This perpetuates their financial insecurity and consequently their lack of headspace for parenting.
		↑ Family distress	
		↓ Caretakers’ headspace	
		↓ Access to social services	**Caretakers who have little trust in government institutions, often grounded in past negative experiences or stories they have heard about bureaucratic hurdles or fear of their child being taken into youth care, are less likely to seek social services and preventive care. This avoidance can worsen their financial security, which in turn reinforces their distrust of these institutions, creating a negative cycle of lessened livelihood security.**
		↓ Livelihood security	
	R2	↓ Caretakers’ trust in government institutions	​
			**Access to social services and preventive care, such as informal aid, community centres, other support programs, or personal social networks provide caretakers with practical and emotional support, making them feel less isolated in the challenges of parenting and better able to support one another. As a result, there is less distress within the family, giving caretakers more mental space to care for their children. With this increased mental space, caretakers are also better able to find and utilize social services, such as participating more actively in community centre activities or seeking preventive care in a timely manner. This creates a reinforcing cycle where access to social services increasingly contributes to the wellbeing of families.**
		↓ Access to social services and preventive care	
		↓ Livelihood security	
		↓ Caretakers’ trust in government institutions	
	R3	↑ Access to social services and preventive care	​
			**Especially when caretakers are not proficient in the Dutch language, it is difficult for them to seek help. When caretakers’ proficiency in the Dutch language improves, they are better able to communicate with others in their environment. This leads to more social interaction in the neighbourhood, such as conversations with neighbours, participation in community activities, or cooperation on common goals. This increased interaction fosters social cohesion in the neighbourhood. Stronger social cohesion contributes to an improved support system for families. All of this, in turn, strengthens caretakers’ language skills, as they have more opportunities to practice and use Dutch in a practical, social context.**
		↑ Family support system	
		↓ Family distress	
		↑ Caretakers’ headspace	
		↑ Access to social services and preventive care	
	R4	↑ Caretakers’ proficiency in the Dutch language	
		↑ Neighbourhood social interaction	
		↑ Neighbourhood social cohesion	
		↑ Family support system	
		↑ Caretakers’ proficiency in the Dutch language	
Tailored education	R1	↑ Atmosphere and structure at school	**When the atmosphere and structure at school are positive (for example, a class where bullying does not occur), preadolescents are more likely to exhibit pro-social behaviour. This pro-social behaviour makes it easier for teachers to build a good and trusting relationship with their students. Thanks to this stronger bond, teachers can focus more on topics such as health and wellbeing during lessons. A school policy on mental health and wellbeing can foster a better atmosphere and structure by for instance letting preadolescents engage in more play, sports and fun activities (e.g., frequent sport sessions during and after school) and focus less on attaining academic skills (but also on e.g., interpersonal and creative skills).**
		↑ Pro-social behaviour	
		↑ Teacher-child relationship quality	
		↑ School policy for health and mental wellbeing	
		↑ Atmosphere and structure at school	Moreover, a positive school climate encourages more pro-social behaviour among preadolescents, which strengthens peer relationships. As a result, incidents of bullying decrease, further enhancing the positive school climate.
	R2	↑ Atmosphere and structure at school	
		↑ Pro-social behaviour	
		↑ Peer relationship quality	
		↓ Bullying, peer conflict and peer pressure	
		↑ Atmosphere and structure at school	

*Note.* The subsystems, feedback loops and associated descriptions that are newly based on the multi-actor perspective are presented in bold. The rest comes from the earlier published science-based CLD. Loops are either reinforcing (R) or balancing (B).

Together, the identified mechanisms illustrate how broad societal forces, such as individualism and digitalization, intersect with daily life to shape preadolescents’ wellbeing. They capture, for instance, the tension between universally high academic expectations and the very uneven home and school conditions that enable success; the pervasive pull of screen-based activities in a 24/7 connected world; the degree to which safe, welcoming neighborhoods foster or inhibit outdoor play and social cohesion; and families’ struggles to navigate complex social‐service systems when under financial stress.

### System function: Archetypes

In total, seven systems archetypes were identified from the mechanisms. Table 3 provides an explanation and illustration of each archetype along with the mechanism on which it is based. The archetypes included two instances of “Success to the Successful”, two of “Shifting the Burden”, two of “Fixes that Fail”, and one of “Drifting Goals”. These archetype descriptions serve to illustrate the system dynamics that are shaped by the identified factors, loops and mechanisms.

**Table 3.** System-based analysis using archetypes.

Subsystem	Mechanism	Archetype	Figures
Increasing pressure & Tailored education	The belief that life is largely controllable through one’s effort – the focus on reaching a ‘perfect picture’ – shapes many individual societies. At school, this translates into high expectations from parents, teachers, peers, and youngsters themselves. Many try to live up to an ideal image: being smart, athletic, popular, and performing well academically. Failing to reach certain goals while others succeed is often seen as a personal failure rather than the result of unequal circumstances. However, actual performance is shaped not only by personal effort but also by the atmosphere and structure at school and at home.	**Success to the successful: Unequal opportunities in performing well academically** Children from households with a higher socioeconomic position (SEP) acquire more resources at home and in school and therefore have more likelihood of succeeding academically (R1) than children from households with a lower SEP (R2), who typically lack such resources. These opportunities – or lack thereof – are reinforced over time, exacerbating the preexisting gap in resources between group A and B. Examples of resources are emotional support and cognitive stimulation by caretakers, parental homework assistance, teachers’ positive expectations and assistance, and the differentiation and early selection of the Dutch educational system.	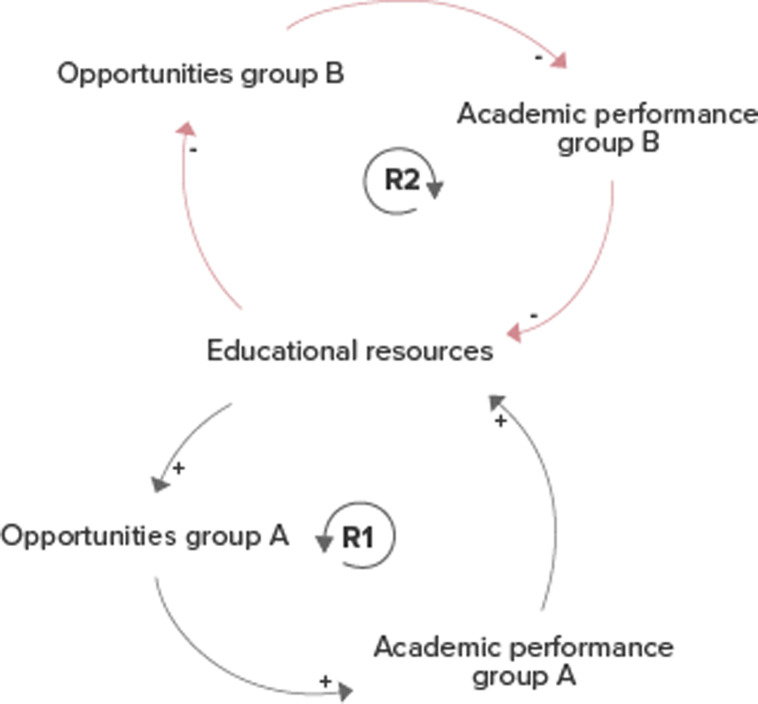
Always online	In a society where continuously being connected with the world around you is the norm, screen use has become deeply embedded in the daily lives of preadolescents. Smartphones play a central role in maintaining such social relationships and to feel included. Not having a smartphone is akin to being left out. At the same time, tech companies continuously develop features that further increase the appeal and pull of screen-based activities. As screen time rises, concerns about its impact (on sleep, concentration, and physical activity) grow as well. In response, attempts to reduce screen use often focus on setting rules or teaching children to self-regulate, aiming to counterbalance the strong pull of a 24/7 connected world.	**Shifting the burden: Societal norms underlying preadolescent smartphone usage** Smartphone use among 9- to 12-year-olds is rising rapidly. Current actions to inhibit smartphone use often target individuals – teaching caregivers to set rules and children to use technology responsibly – offering only short-term reductions (B1). This shifts responsibility away from actions targeted at the society or business level (B3), such as collectively delaying the first smartphone age or regulating addictive algorithms, which would be more long-term solutions (B2). Without such systemic actions, tech companies will keep creating products that individual efforts cannot counter.	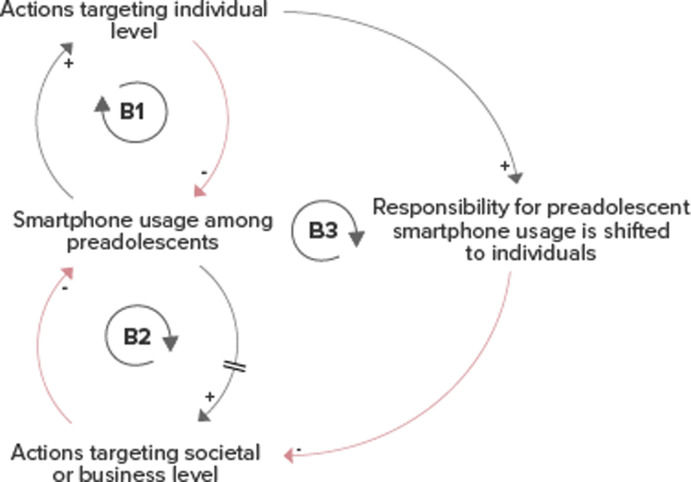
Always online & Caretaker challenges	In many families, caretakers try to limit their children’s screen time by setting rules about when, where and how long screens can be used. However, the impact of these rules largely depends on the quality of the parent‒child relationship. When the connection is strong and based on mutual trust, rules are more likely to be accepted and followed. However, when the relationship is tense or inconsistent, rules may be met with resistance or ignored altogether. In addition, children often observe their parents' own screen behavior, such as using phones for work, relaxation, or out of habit, and this strongly shapes what children see as normal or acceptable behaviour.	**Fixes that fail: Caretakers as role models** Smartphone use among 9- to 12-year-olds is rising rapidly. In response, many parents and caretakers introduce rules to limit screen time, which initially reduces usage (B1). However, when role models do not follow these rules themselves and continue to use their own phones frequently (because that’s normal), a gap emerges between the rules they set and the behaviour they demonstrate (R1). Preadolescents tend to imitate the behaviour of their role models, and this inconsistency increases their resistance to follow the rules and reduce their screen time. Over time, the combination of strict rules and contradictory role-model behaviour leads to a rebound effect: smartphone use among preadolescents increases.	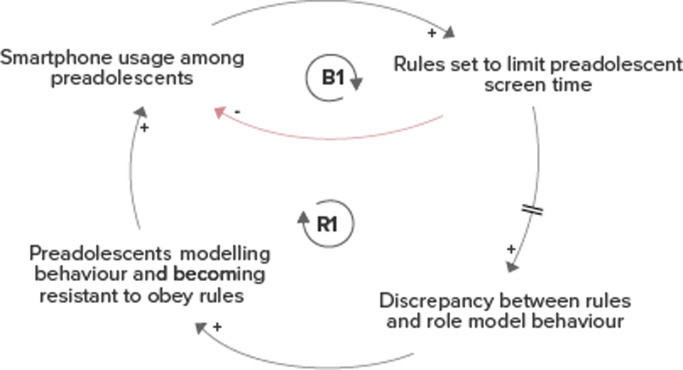
Neighbourhood ties	The quality and safety of a neighbourhood strongly influence how often preadolescents play outside. When outdoor spaces are perceived as safe, well-maintained, and welcoming, children are more likely to spend time outdoors and engage in social and physical activities. Conversely, unsafe neighbourhoods with lacking facilities to play diminishes the prevalence of outdoor play. This reduction in outdoor activity limits opportunities for social interaction, physical exercise, and positive development.	**Drifting goals: Availability of playgrounds and sports facilities in the neighbourhood** Cities face limited resources to facilitate sufficient housing and at the same time invest in the quality of living via e.g. community playground facilities. In current times of housing shortages, there is a higher political pressure to invest in new housing projects than e.g., to create attractive sports and play facilities in neighbourhoods (B1). This lack of priority erodes the original goal of creating high-quality child-friendly, safe and accessible spaces. Instead, playgrounds and sports facilities are now often relocated to the edges of the city to make room for new housing. Accessibility and usage consequently decline, further eroding the feeling of priority to have them.	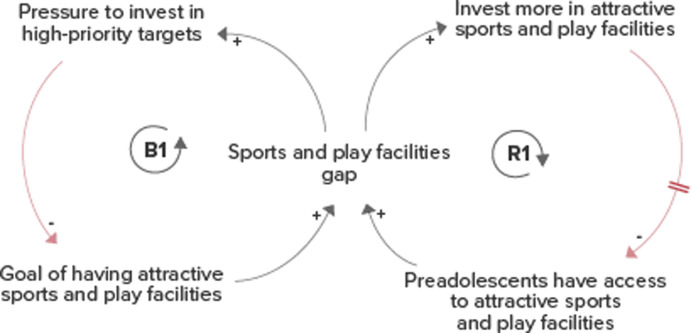
**Supportive bonds & Neighbourhood ties**	Due to the individualistic nature of society, social cohesion in neighbourhoods declines. Families are more focused on themselves and less on one another. As a result, they are less likely to form a safety net for each other, even though an informal support system is key for social support and limiting distress.	**Shifting the burden: Informal support** In the Netherlands, formal care is readily accessible, allowing families and preadolescents in distress (e.g. (mental) health crises, financial problems) to receive professional support quickly (B2). While informal support networks might alleviate and even prevent distress, the once-common norm of 'it takes a village to raise a child' has faded in an age of growing individualism (B2). Relying on professional help may alleviate problems in the short term, but over time it weakens social cohesion, increases dependence on external formal support, and erodes trust in informal networks (B3)	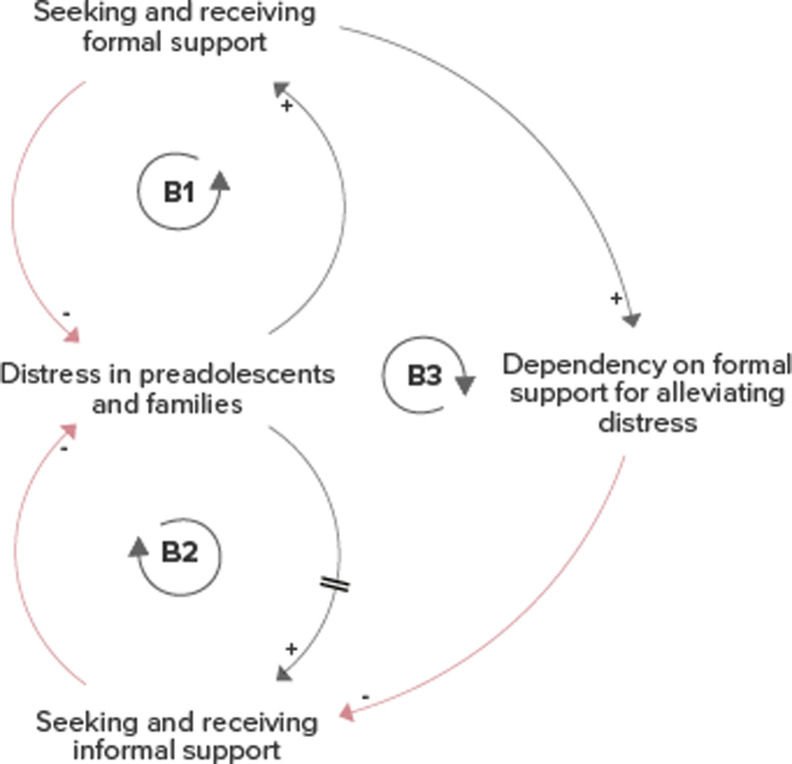
**Lost in the system & Safety and security**	Families in vulnerable financial situations can easily become lost in the complex web of social services and preventive care regulations. When a family's livelihood becomes less secure - due to financial problems or unemployment, for example - they experience intense distress to meet their basic needs. This increased distress reduces caretakers’ headspace. As a result, it becomes harder for them to focus on caring for their children or finding help, especially when access to social services and preventive care is complicated. This further worsens their situation, putting even more strain on the family's livelihood.	**Fixes that fail: Fragmented support for poverty** Various organizations and municipal departments in Amsterdam develop plans to combat livelihood insecurity. Each of these plans addresses a different symptom of poverty, ranging from energy vouchers and subsidies for children's sports and cultural activities to waivers on municipal taxes, reducing livelihood insecurity in the short run (B1). However, this increased multitude of rules and support schemes reduces clarity, causing many families to miss out on available assistance. This problem is especially pronounced for families who have previously had negative experiences with government institutions, and, for example, fear fines or struggle with the Dutch language. As a result, these well-intentioned support options can backfire: the complexity discourages uptake, leading vulnerable families to miss out on aid and experience more livelihood insecurity (B2).	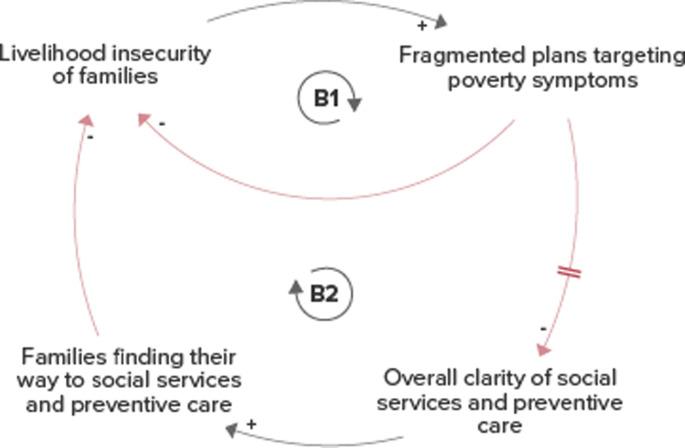
**Tailored education**	Preadolescents spend much of their time at school. A positive relationship with a teacher, opportunities for talent and identity development, parental involvement in school, and school policies/programs that focus on health and mental wellbeing are all crucial for strong academic performance and good mental health.	**Success to the successful: Schools, goals and limited capacity** Well-resourced schools are easily able to implement programs that support health and wellbeing, such as the Amsterdam programs “Familie School” (‘Family school’) or “Gezonde School” (‘Healthy school’), because they have the capacity (financial resources, hours) for applying and implementing. Such programs bring extra resources to a school (R1). Underresourced schools, already stretched by basic demands, struggle to access these opportunities (R2). As a result, schools that are doing well keep advancing, while schools with fewer resources fall further behind in creating supportive environments for all students.	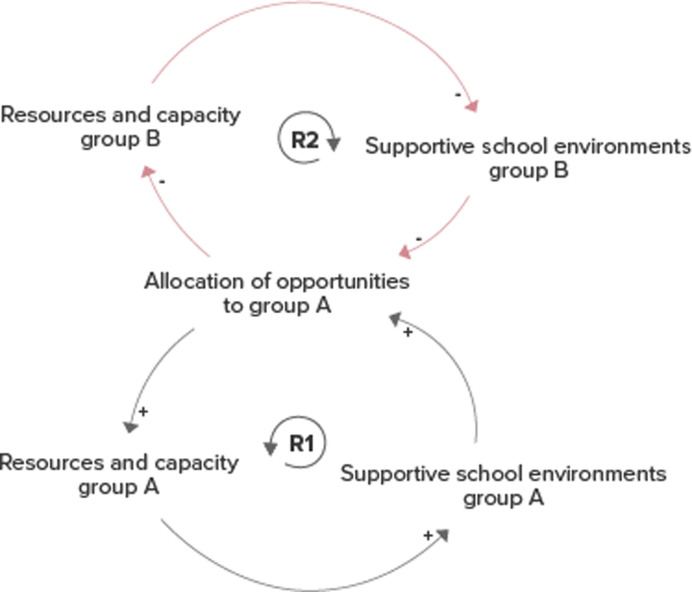

For example, the analysis of the system’s structure (Table 2) revealed a mechanism around caretakers requiring sufficient mental headspace to access social services and preventive care. The archetypal pattern explaining why mental headspace is a prerequisite for seeking services is a “Fixes that Fail” for vulnerable families. That is, numerous short-term plans and regulations (‘fixes’) aim to improve livelihood security for vulnerable families, but their cumulative effect creates long-term complexity and reduced visibility. Consequently, these well-intentioned support measures can backfire: the resulting complexity discourages uptake, leaving vulnerable families without sufficient headspace unable to access aid and ultimately experiencing greater livelihood insecurity.

## Discussion

This study aimed to gain a richer understanding of the system dynamics underlying preadolescent mental wellbeing. Unlike earlier positivist approaches that focused narrowly on a single relationship or determinant, this study did not provide an exhaustive account of detailed evidence, but rather aimed to explore both the interrelations between factors and feedback loops (the system’s structure) and the patterns that underlie these interactions (the system’s behaviour) at the population level.

### System structure: Multi-actor CLD

The multi-actor CLD included 62 factors, 50 feedback loops, and 11 subsystems. The multi-actor perspective adds two subsystems, seven feedback loops, and nine factors to the original science-based CLD. By integrating the perspective of stakeholders with the scientific perspective, the CLD captures both proximal and distal factors and their interconnections, which were then used to identify larger mechanisms. Many of these new mechanisms illustrate indirect influences, such as caretakers not finding their way to preventive services and care or providing in-depth knowledge of a specific theme, such as adultification, which interferes with preadolescent identity development.

Moreover, the multi-actor perspective highlights that while the system’s structure captures dynamics across a broad range of themes, the specific expression of each mechanism depends on the social and environmental context. For example, neighbourhood quality and safety influence children’s outdoor play in different ways in different contexts: in one area through traffic or distant playgrounds or in another through (drug-related) crime. While the outcome – children spending too much time indoors – remains the same, the specific underlying mechanisms vary. Similarly, mechanisms differ across cultures. For example, in some collectivist cultures, mental health issues are seen as private family matters, with the home considered a closed space and discussing personal or emotional problems with outsiders viewed as inappropriate. These examples illustrate that while system-level mechanisms are broadly applicable, understanding the nuances of neighbourhood contexts and cultural values is essential for accurate interpretation. This interpretative nature is particularly important to consider when discussing the system’s structure with stakeholders.

### System’s behaviour: Archetypes

Systems archetypes, although introduced by Senge in 1990, have been used mainly in empirical contexts such as biodiversity and agriculture.^27–29^ This study is among the first to use them to understand a complex social issue. Among the multitude of interconnected factors, loops, and mechanisms, seven archetypes were identified that together provide an understanding of the system’s behaviour.

The seven archetypes predominantly reflected macrolevel social or political phenomena. Interestingly, one archetype (‘*Fixes that fail: Caretakers as role models’*) was more at the micro level, describing how caretakers' modelling behaviour is key when setting rules for preadolescents’ smartphone usage. When thinking about actions that could disrupt the patterns described there, another archetype (‘*Shifting the burden: Societal norms underlying preadolescent smartphone usage’*) comes to mind. That is, actions targeting the role modelling behaviour of caretakers place responsibility at the individual level instead of at the societal or business level. Ultimately, combining these insights when developing actions is important. Actions focused on the societal or political level, such as shifting social norms regarding the age at which children receive their first smartphone or regulating the addictive algorithms used by tech companies, may have the greatest impact, but could be supplemented with subactions focused on the individual level, such as stimulating positive role model behaviour.

These identified patterns are supported by insights from previous literature. The first pattern (‘*Unequal opportunities in performing well academically*’) is consistent with research showing that socioeconomic status is positively associated with cognitive performance^30^ and that small initial differences can amplify over time through cumulative advantage mechanisms.^31^ For the second pattern (‘*Societal norms underlying preadolescent smartphone usage*’), research has shown that adolescent technology use is largely driven by social pressure,^32^ indicating the need for coordinated societal efforts to establish new norms.^33^ Third, previous research highlights the importance of parental modelling and suggests targeting both children’s and caretakers’ behaviour in interventions,^34^ in line with the pattern *of ‘Caretakers as role models*’. The fourth pattern (*‘Availability of playgrounds and sports facilities*’) can be linked to research demonstrating that budget cuts in the Netherlands have led many municipal governments to scale down their investments in sport facilities, despite researchers calling for increasing the attractiveness of sport facilities.^35^ With respect to the fifth pattern (‘*Informal support’*), Dutch youth policy tends to treat informal networks as add-on tasks rather than essential foundations of care,^36^ despite evidence that such networks enhance mental wellbeing.^37^ The sixth pattern (‘*Fragmented support for poverty’*) is consistent with Dutch studies reporting that fragmented rules, opaque eligibility, and short-term interventions hinder families’ access to effective support.^38–40^ Finally, policy reports have highlighted that schools with strong leadership, parental involvement, and stable staff are more likely to implement sustainable interventions,^41^ reflecting the seventh pattern *‘Schools, goals and limited capacity’*.

### System structure versus behaviour: Findings and implications

Overall, the multi-actor CLD mapped the system’s structures that shape preadolescent mental wellbeing. It provides insight into the relationships between multiple factors, highlighting not only surface-level symptoms but also “causes behind the causes.” The systems archetype analysis then provided an extra layer of insight into the underlying patterns sustaining a certain mechanism, e.g., the fragmented landscape of rules and schemes for livelihood security support, explaining why caretakers need mental headspace to gain access.

This analysis of the system’s structure and behaviour reveals several insights. First, the analysis identified many distinct but interconnected layers of dynamics, ranging from immediate pressures in school or family life to broader societal norms and institutional structures. These insights reveal how there is no easy explanation or quick fix for this complex public health issue.

Second, a number of societal norms or paradigms that were embedded within system dynamics were identified. These include the belief that success is self-made and must be rewarded; the perception that children from higher socioeconomic backgrounds are more ‘talented’; the expectation of being constantly online and reachable; the emphasis on individual responsibility in dealing with technological challenges; and the persistent stigma around discussing mental health issues.

These systems understanding insights can inform meaningful action, empowering policymakers, educators, and communities to disrupt patterns (the identified archetypes) that negatively impact preadolescent mental wellbeing. That is, viewing complex public health issues through a systems lens can encourage practitioners and policymakers to (1) adopt a systems thinking perspective that moves beyond isolated symptoms to consider the underlying structures, goals, and belief systems sustaining these challenges^42^ and (2) engage in adaptive action, embracing real-time learning and iterative responses that address deeper structural drivers.^43^

### Strengths, limitations, and future directions

A strength of this study lies in its novel approach to analyse both the system’s structure and behaviour, showing the interconnected layers of dynamics underlying preadolescent mental wellbeing, as well as the archetypes sustaining the system. Moreover, the use of a science-based CLD ensures both theoretical consistency and contextual relevance. In addition, the large volume of qualitative data, combined with team-based analysis, enhanced the credibility of the findings.

This study has several limitations. The methods used, such as *The Tree*, were cocreated with stakeholders and remain partially experimental. While we believe that this participatory approach enables high levels of engagement and inclusivity, it also introduces elements whose validity has yet to be thoroughly established. For example, to understand the perspective of preadolescents, we relied on trusted professionals’ interpretations. Although this ensured that these young teenagers spoke about potentially sensitive topics with a trusted professional, this added an extra layer of interpretation. Nevertheless, the consistency across neighbourhoods, shared perspectives among different respondent groups and alignment with existing scientific literature underscore the robustness of the findings. Moreover, the evolving nature of the codebook during analysis may have introduced an interpretive bias, although this bias was mitigated through interneighbourhood checks, systematic reflection and regular team discussions. A final limitation relates to the systems (archetype) analysis. These analyses are subject to the researchers’ interpretations and could therefore have been unintentionally influenced by the researchers (e.g., by their personal beliefs, presumptions, etc.).^44^ We mitigated this through iterative, reflexive team discussions and, in cases of doubt, targeted member checking with interviewed or external professionals. Future systems research should continue recognizing these archetypal patterns to mitigate these risks of bias.

In the context of the current study, future research should focus on translating systems understanding into systems change. One promising avenue is to investigate whether disrupting the identified systems archetypes can lead to improvements in preadolescent mental wellbeing. In addition, further methodological advancements are needed to refine and validate participatory tools such as *The Tree* to investigate how these tools can be used to better understand system dynamics.

## Conclusion

This study provides an understanding of the system dynamics underlying preadolescent mental wellbeing in Amsterdam. By integrating multiple perspectives and analysing the system as a whole, through developing a multi-actor CLD and conducting a systems archetype analysis, it becomes clear that the dynamics underlying preadolescent wellbeing are wide-ranging in terms of themes, spanning a broad range without being limited to a specific domain. In many dynamics, social norms revolving around, for instance, individualism or the 24/7 online world play a central role. Together, the findings highlight a critical need to shift from reactive, short-term solutions toward more cross-domain, preventive, structural strategies. These systems archetypes may serve as powerful tools to catalyse change. By viewing complex challenges through a ‘systems (archetype) lens’, policymakers, educators, and communities can better understand the bigger picture and work together to create more resilient and inclusive environments that support the mental wellbeing of preadolescents.

## Supplemental material

Supplemental material - System dynamics of preadolescent mental wellbeing: A multi-actor perspective in Amsterdam using system archetypes Supplemental material for System dynamics of preadolescent mental wellbeing: A multi-actor perspective in Amsterdam using systems archetypes by Eline M. Meuleman, Vincent Busch, Wilma E. Waterlander, Nanda E. van der Poel, Carry M. Renders, and Maartje M. van Stralen in Journal of Public Health Research.

## Data Availability

The data that support the findings of this study are available from the corresponding author upon reasonable request.*
