# Erratum to “System dynamics of preadolescent mental wellbeing: A multi-actor perspective in Amsterdam using systems archetypes”

**DOI:** 10.1177/22799036261472551

**Published:** 2026-07-29

Meuleman EM, Busch V, Waterlander WE, et al. System dynamics of preadolescent mental wellbeing: A multi-actor perspective in Amsterdam using systems archetypes. J Public Health Res 2026; 15(2): 1–23. doi:10.1177/22799036261455634

In this article, following updates have been made to Tables 2 and 3:

In Table 3, the figures have been replaced with updated versions given below:


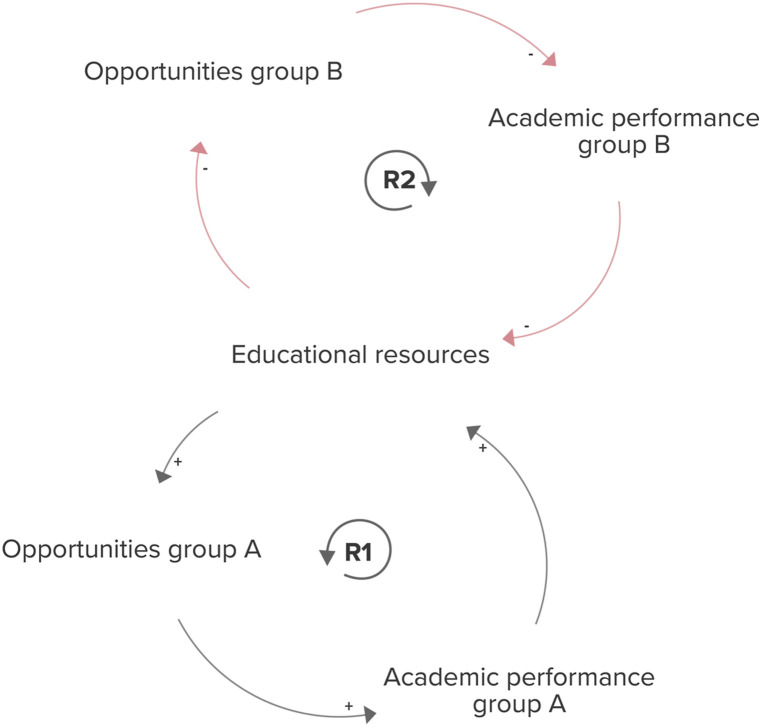



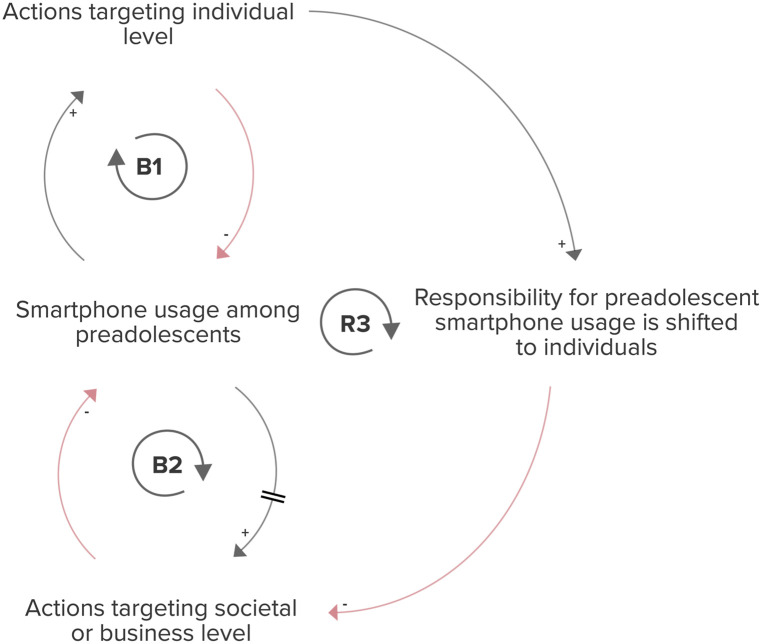



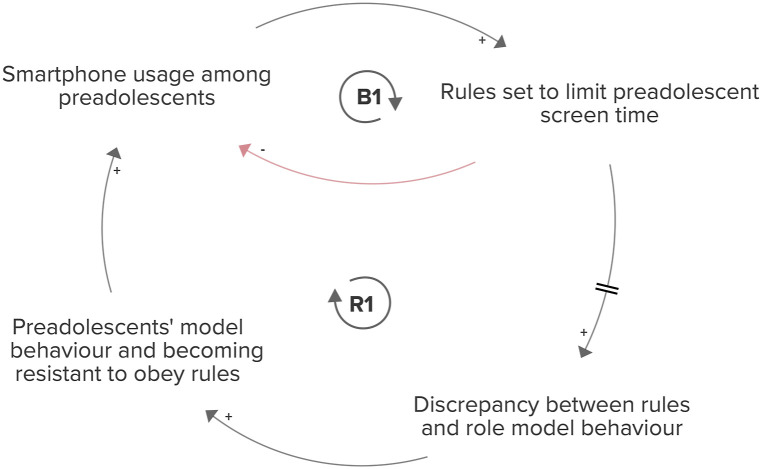



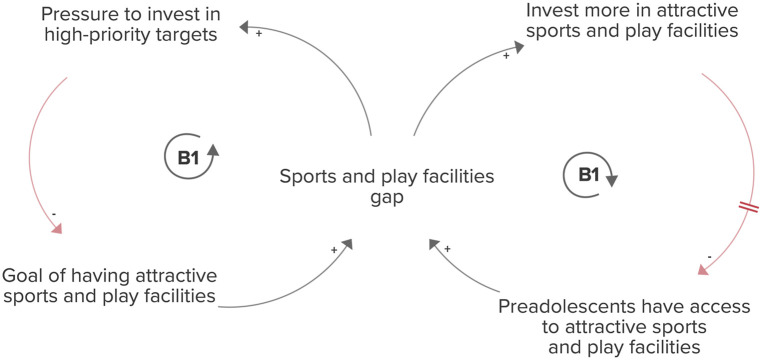



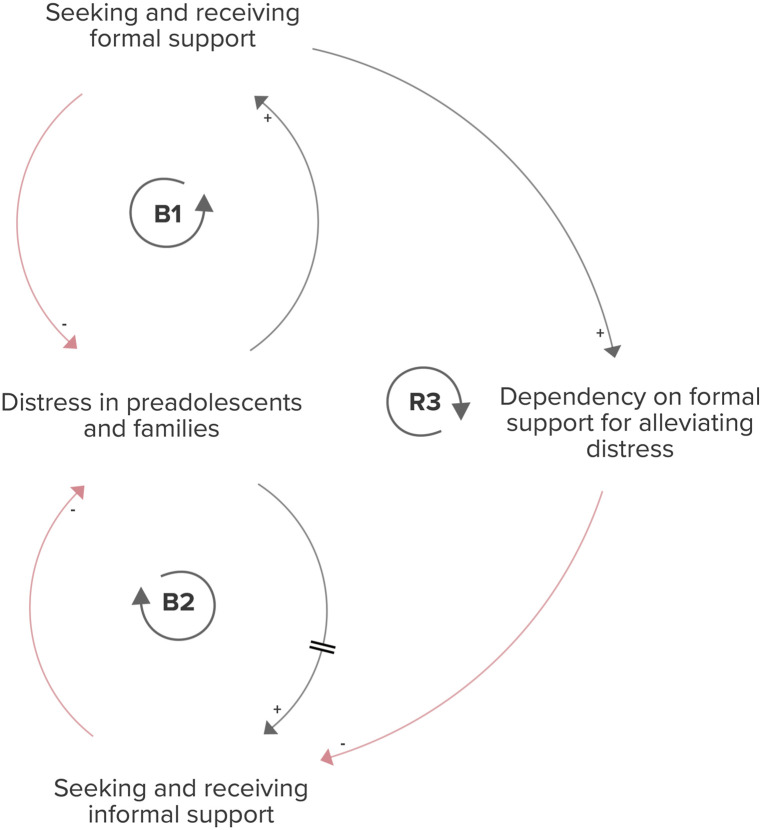



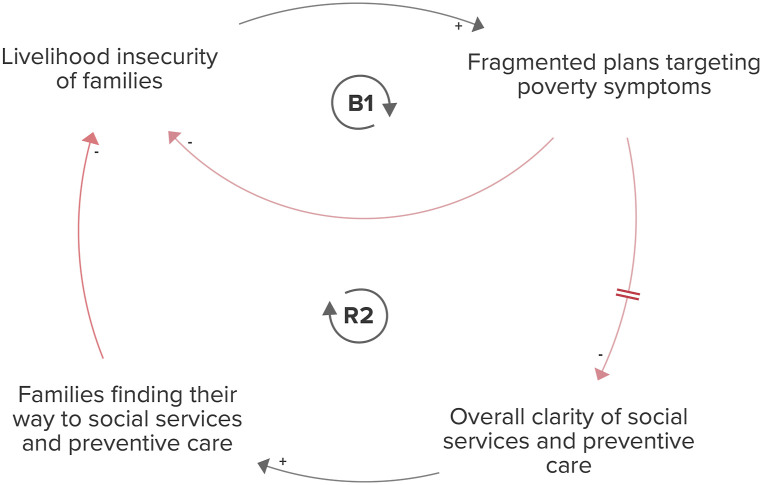



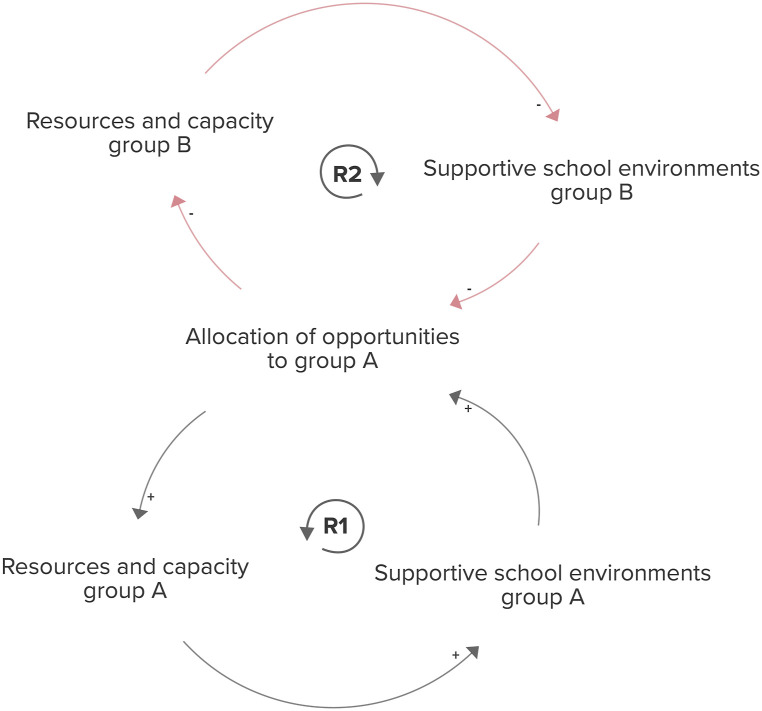


In Table 2, formatting has been updated to remove unnecessary line breaks and spacing, ensuring that the content is displayed correctly.

The online version has been updated accordingly.

