# Supplementary material for: System dynamics of preadolescent mental wellbeing: A multi-actor perspective in Amsterdam using systems archetypes
Source: J Public Health Res. 2026 Jun 24;15(2):22799036261455634. doi: 10.1177/22799036261455634 (PMC13305245; doi:10.1177/22799036261455634)
Supplement: Supplemental material - System dynamics of preadolescent mental wellbeing: A multi-actor perspective in Amsterdam using system archetypes [file sj-pdf-1-phj-10.1177_22799036261455634.pdf]

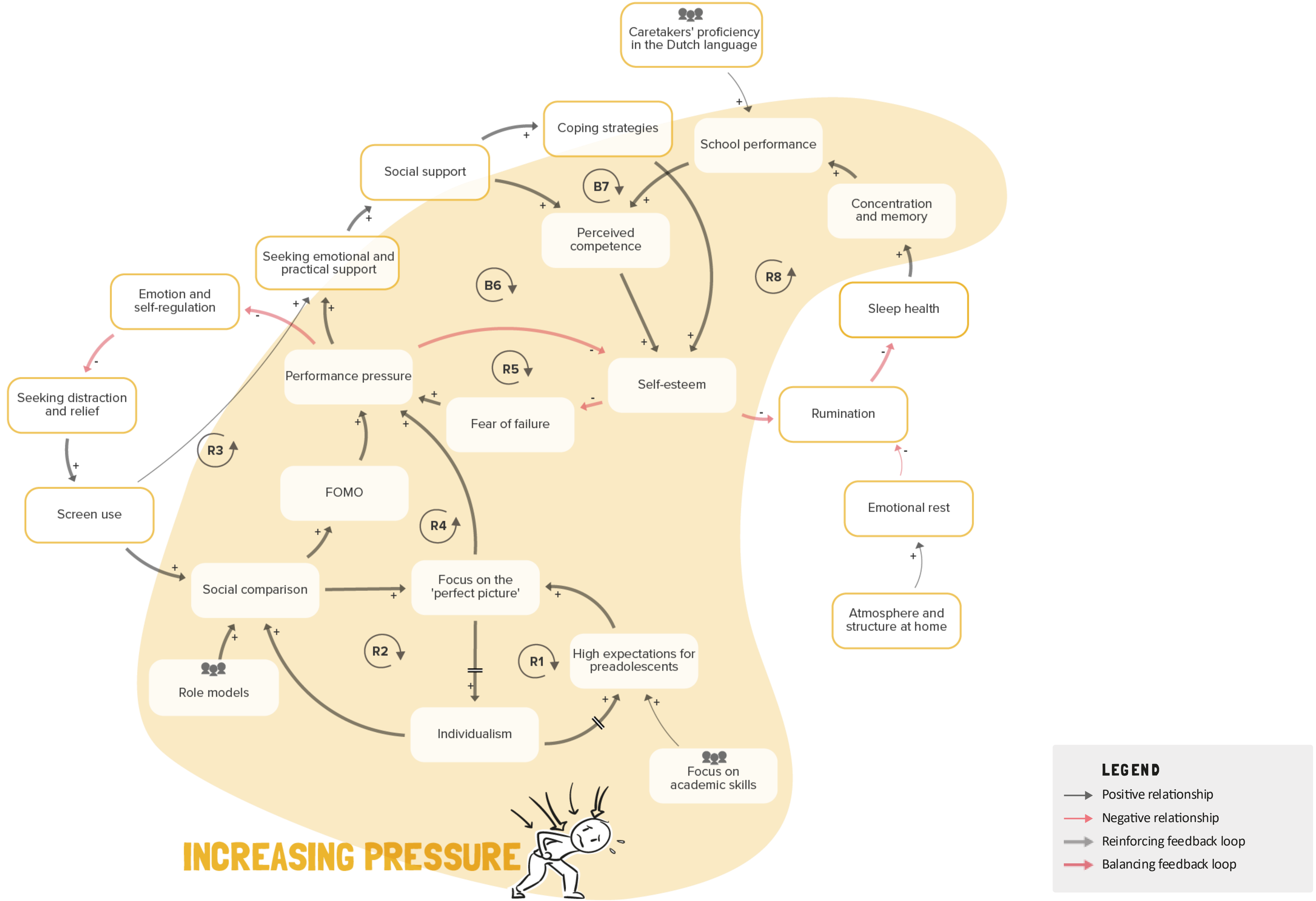
Caretakers' proficiency in the Dutch language
Coping strategies
School performance
Social support
B7
Perceived competence
Concentration and memory
Seeking emotional and practical support
B6
R8
Emotion and self-regulation
Sleep health
Performance pressure
R5
Self-esteem
Seeking distraction and relief
Fear of failure
Rumination
R3
FOMO
Screen use
R4
Emotional rest
Social comparison
Focus on the 'perfect picture'
Atmosphere and structure at home
R2
R1
High expectations for preadolescents
Role models
Individualism
Focus on academic skills
INCREASING PRESSURE
LEGEND
Positive relationship
Negative relationship
Reinforcing feedback loop
Balancing feedback loop

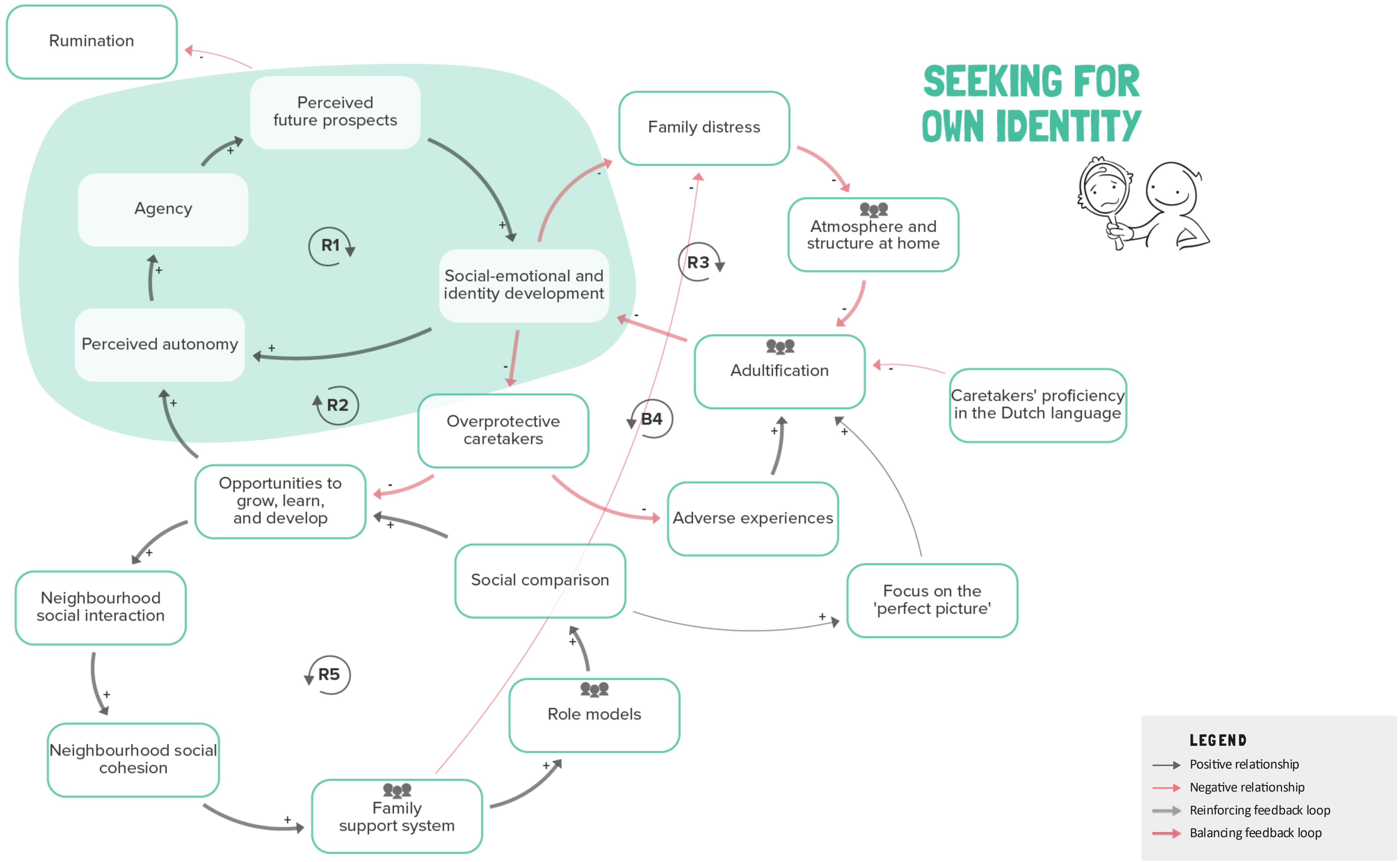

SEEKING FOR OWN IDENTITY
Rumination
Perceived future prospects
Agency
R1
Social-emotional and identity development
Perceived autonomy
R2
Family distress
R3
Atmosphere and structure at home
Adultification
Caretakers' proficiency in the Dutch language
B4
Overprotective caretakers
Opportunities to grow, learn, and develop
Adverse experiences
Social comparison
Focus on the 'perfect picture'
Neighbourhood social interaction
R5
Neighbourhood social cohesion
Role models
Family support system
LEGEND
Positive relationship
Negative relationship
Reinforcing feedback loop
Balancing feedback loop

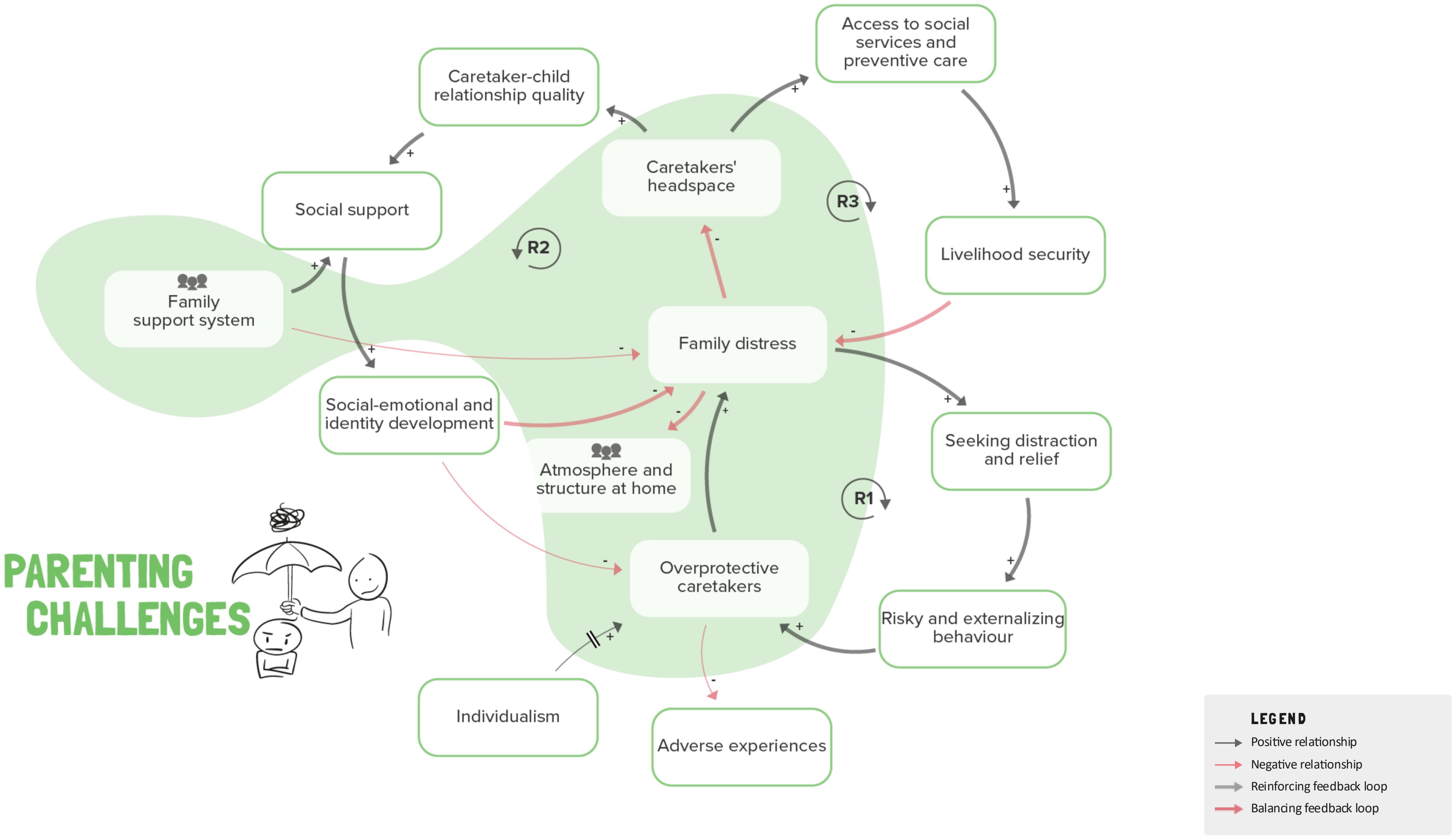

PARENTING CHALLENGES
Caretaker-child relationship quality
Access to social services and preventive care
Social support
Caretakers' headspace
R3
R2
Livelihood security
Family support system
Family distress
Social-emotional and identity development
Seeking distraction and relief
Atmosphere and structure at home
R1
Overprotective caretakers
Risky and externalizing behaviour
Individualism
Adverse experiences
LEGEND
Positive relationship
Negative relationship
Reinforcing feedback loop
Balancing feedback loop

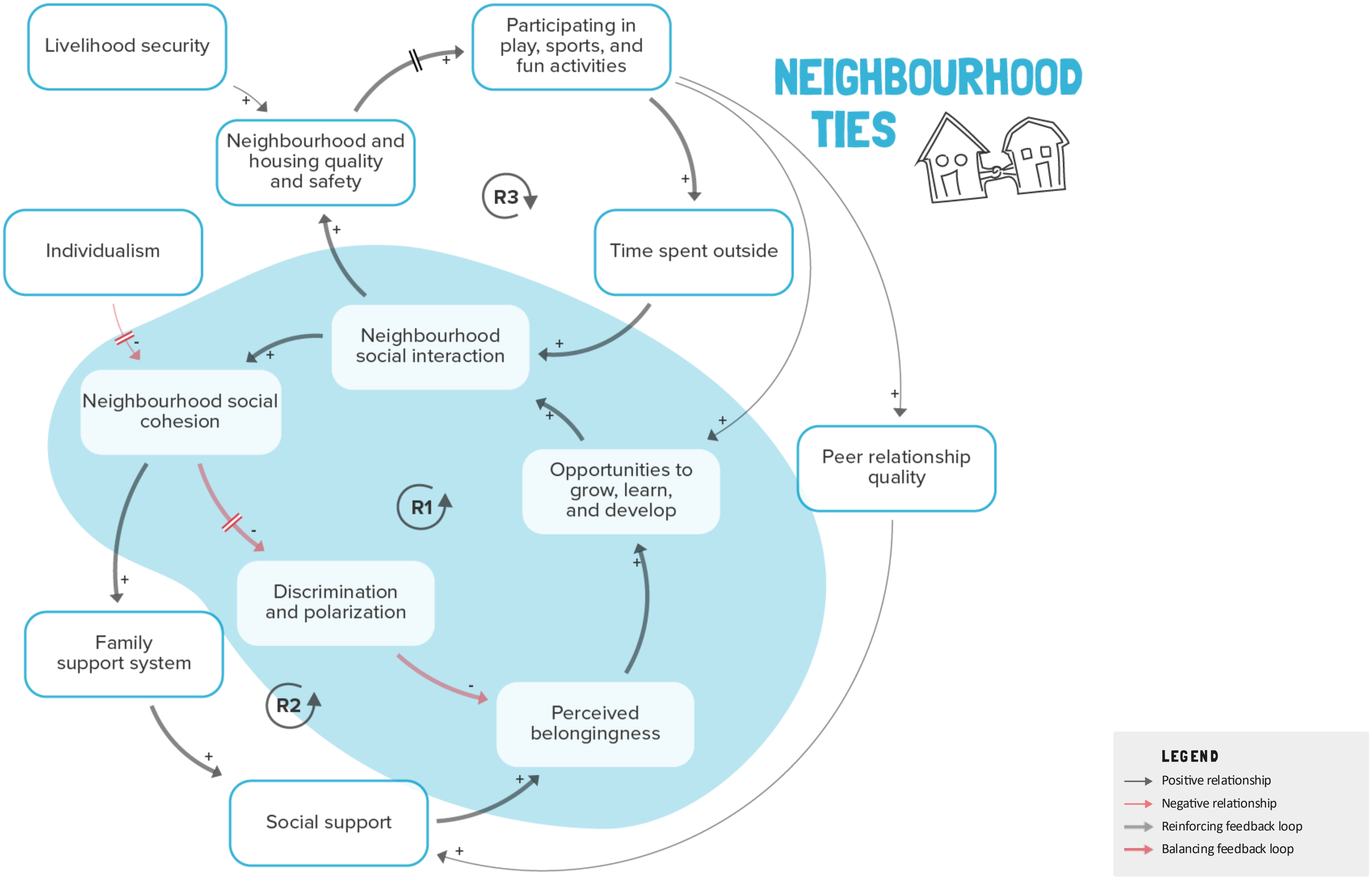
NEIGHBOURHOOD TIES
Livelihood security
Neighbourhood and housing quality and safety
Participating in play, sports, and fun activities
Individualism
Time spent outside
R3
Neighbourhood social interaction
Neighbourhood social cohesion
Opportunities to grow, learn, and develop
Peer relationship quality
R1
Discrimination and polarization
Family support system
R2
Perceived belongingness
Social support
LEGEND
Positive relationship
Negative relationship
Reinforcing feedback loop
Balancing feedback loop

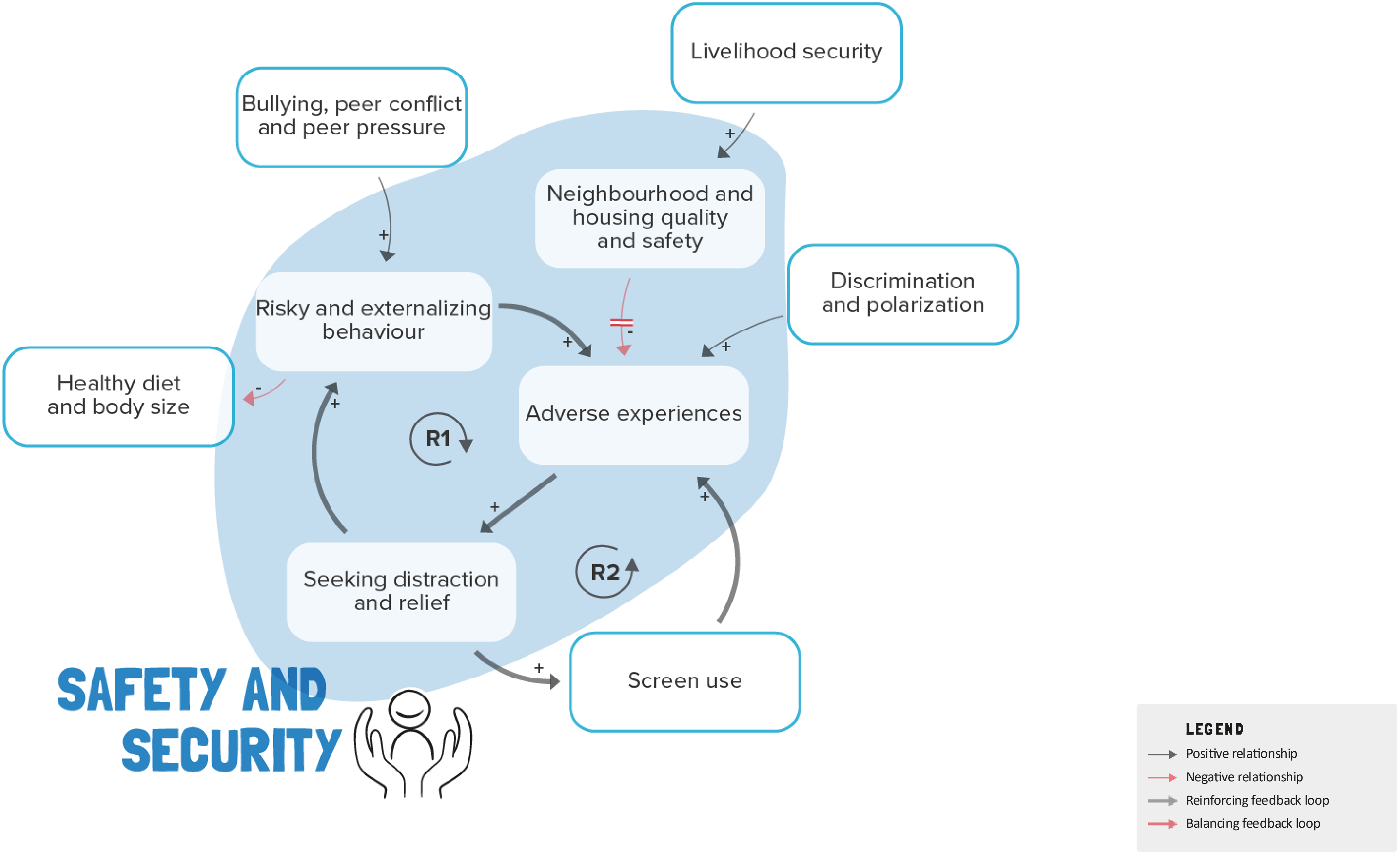
Livelihood security
Bullying, peer conflict and peer pressure
Neighbourhood and housing quality and safety
Discrimination and polarization
Risky and externalizing behaviour
Healthy diet and body size
Adverse experiences
R1
R2
Seeking distraction and relief
Screen use
SAFETY AND SECURITY
LEGEND
Positive relationship
Negative relationship
Reinforcing feedback loop
Balancing feedback loop

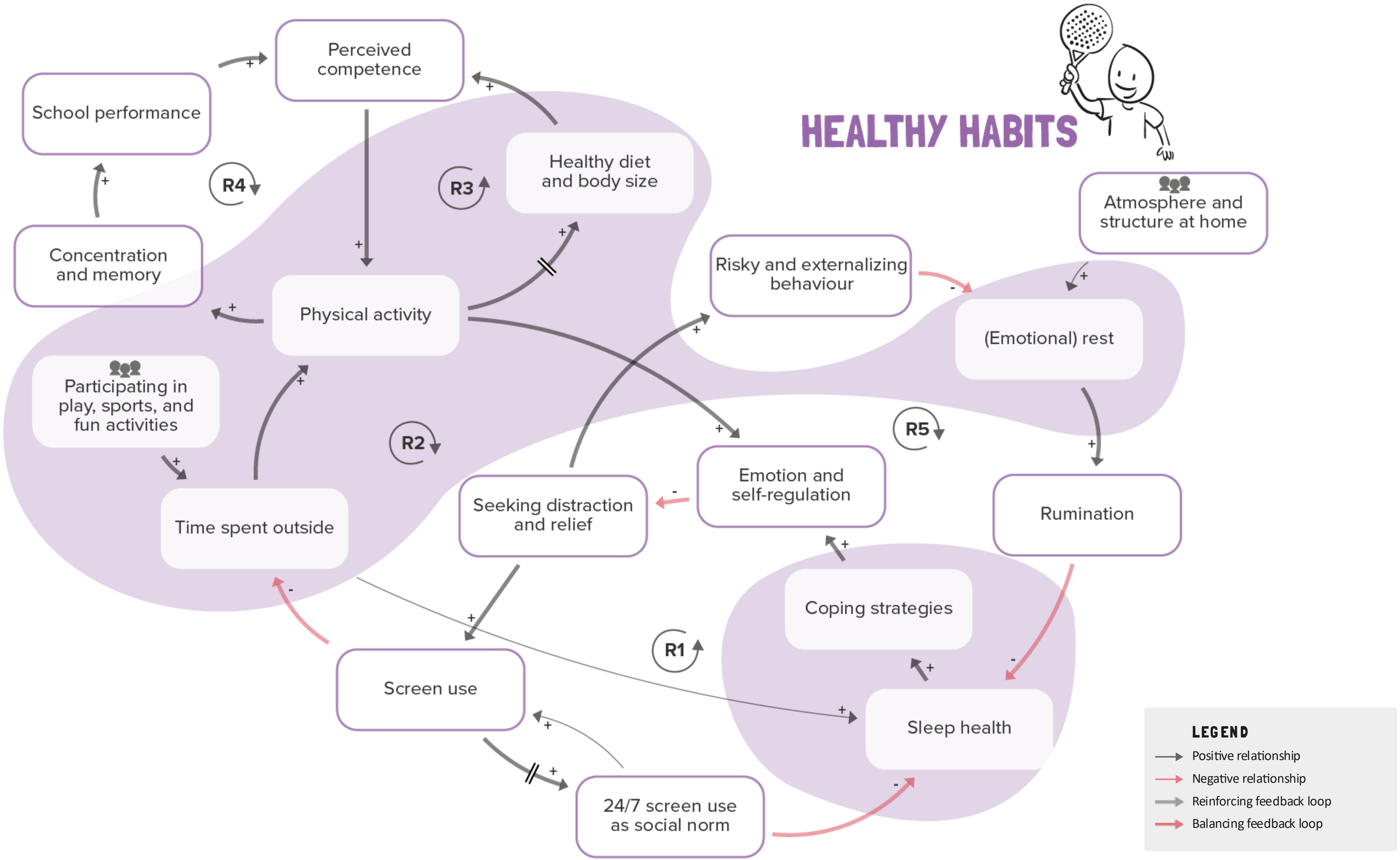
HEALTHY HABITS
School performance
Perceived competence
Healthy diet and body size
Concentration and memory
Physical activity
Participating in play, sports, and fun activities
Time spent outside
Atmosphere and structure at home
Risky and externalizing behaviour
(Emotional) rest
Emotion and self-regulation
Seeking distraction and relief
Rumination
Coping strategies
Screen use
Sleep health
24/7 screen use as social norm
R1
R2
R3
R4
R5
LEGEND
Positive relationship
Negative relationship
Reinforcing feedback loop
Balancing feedback loop

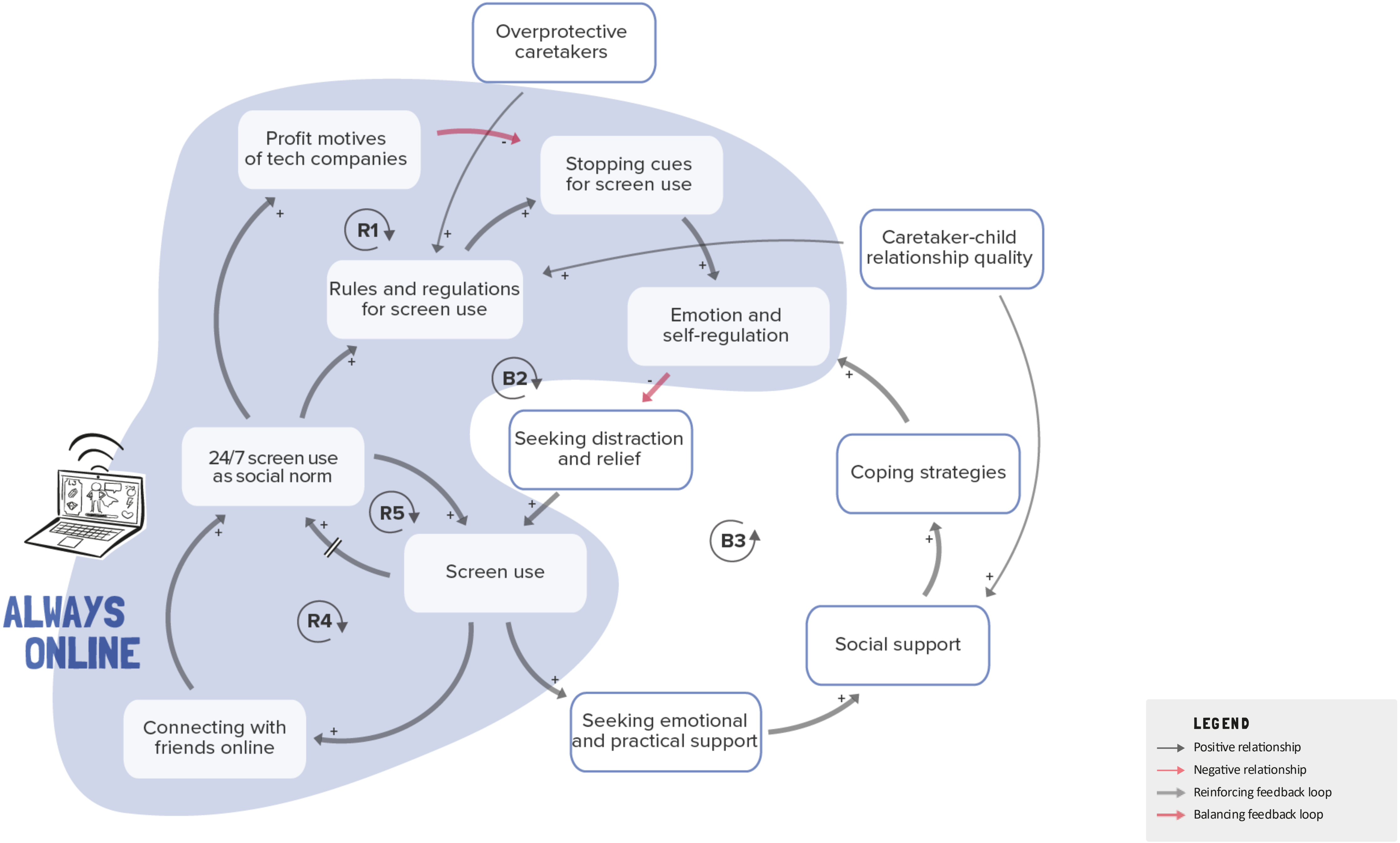

Overprotective caretakers
Profit motives of tech companies
Stopping cues for screen use
Caretaker-child relationship quality
Rules and regulations for screen use
Emotion and self-regulation
R1
B2
Seeking distraction and relief
24/7 screen use as social norm
Coping strategies
R5
B3
Screen use
ALWAYS ONLINE
R4
Social support
Connecting with friends online
Seeking emotional and practical support
LEGEND
Positive relationship
Negative relationship
Reinforcing feedback loop
Balancing feedback loop

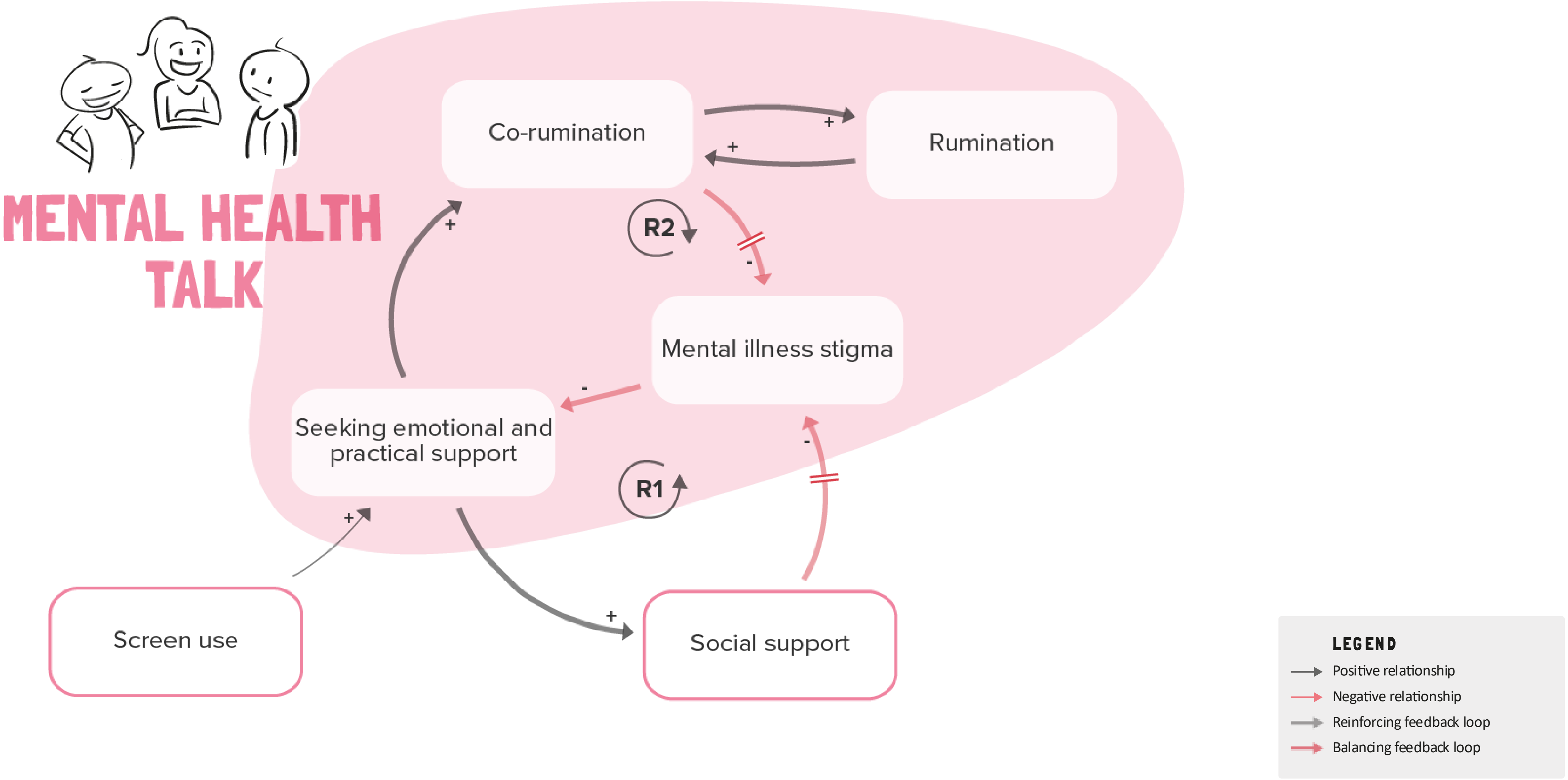
MENTAL HEALTH TALK
Co-rumination
Rumination
R2
Mental illness stigma
Seeking emotional and practical support
R1
Screen use
Social support
LEGEND
Positive relationship
Negative relationship
Reinforcing feedback loop
Balancing feedback loop

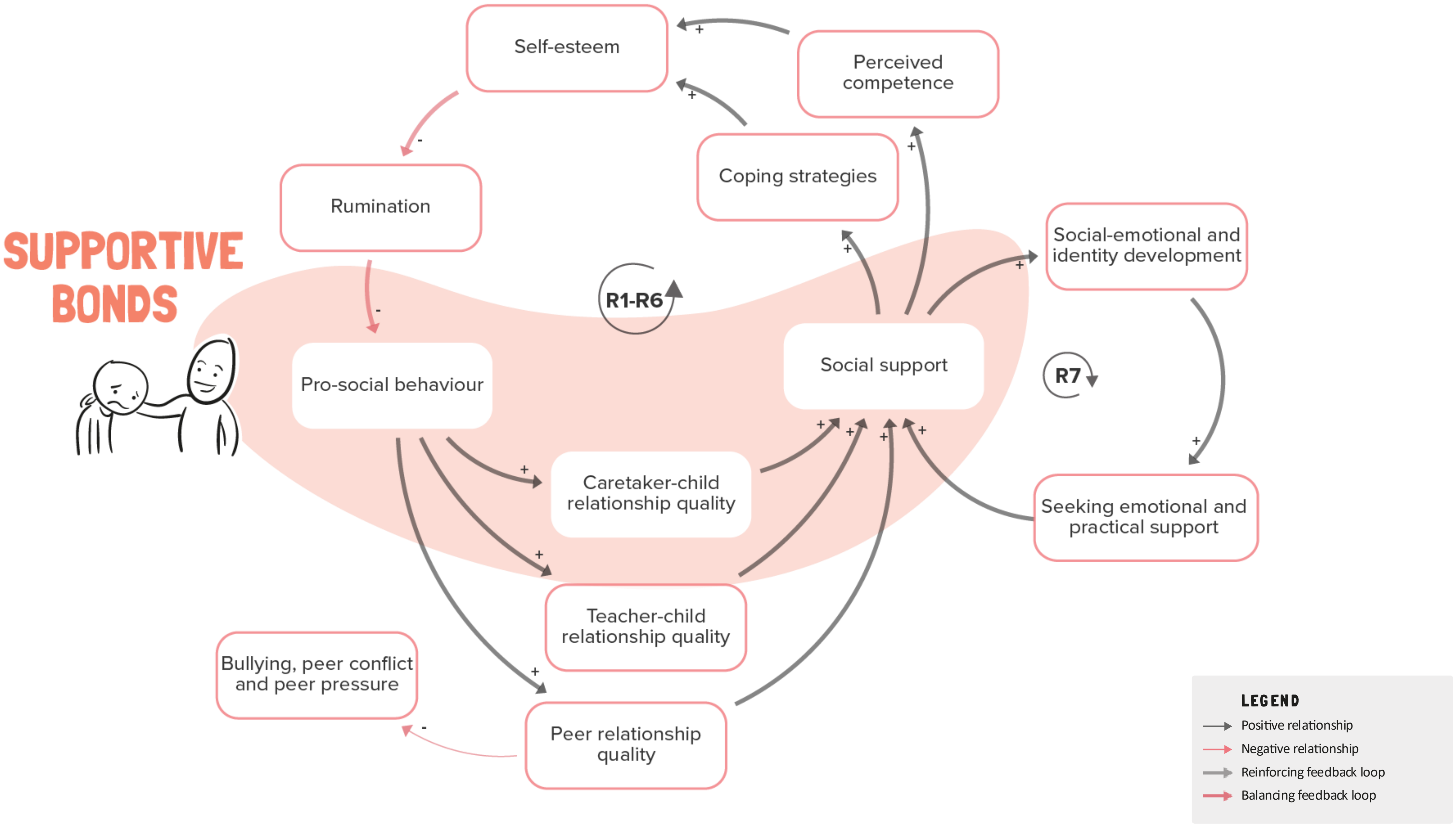

SUPPORTIVE BONDS
Self-esteem
Perceived competence
Rumination
Coping strategies
Social-emotional and identity development
R1-R6
Pro-social behaviour
Social support
R7
Caretaker-child relationship quality
Seeking emotional and practical support
Teacher-child relationship quality
Bullying, peer conflict and peer pressure
Peer relationship quality
LEGEND
Positive relationship
Negative relationship
Reinforcing feedback loop
Balancing feedback loop

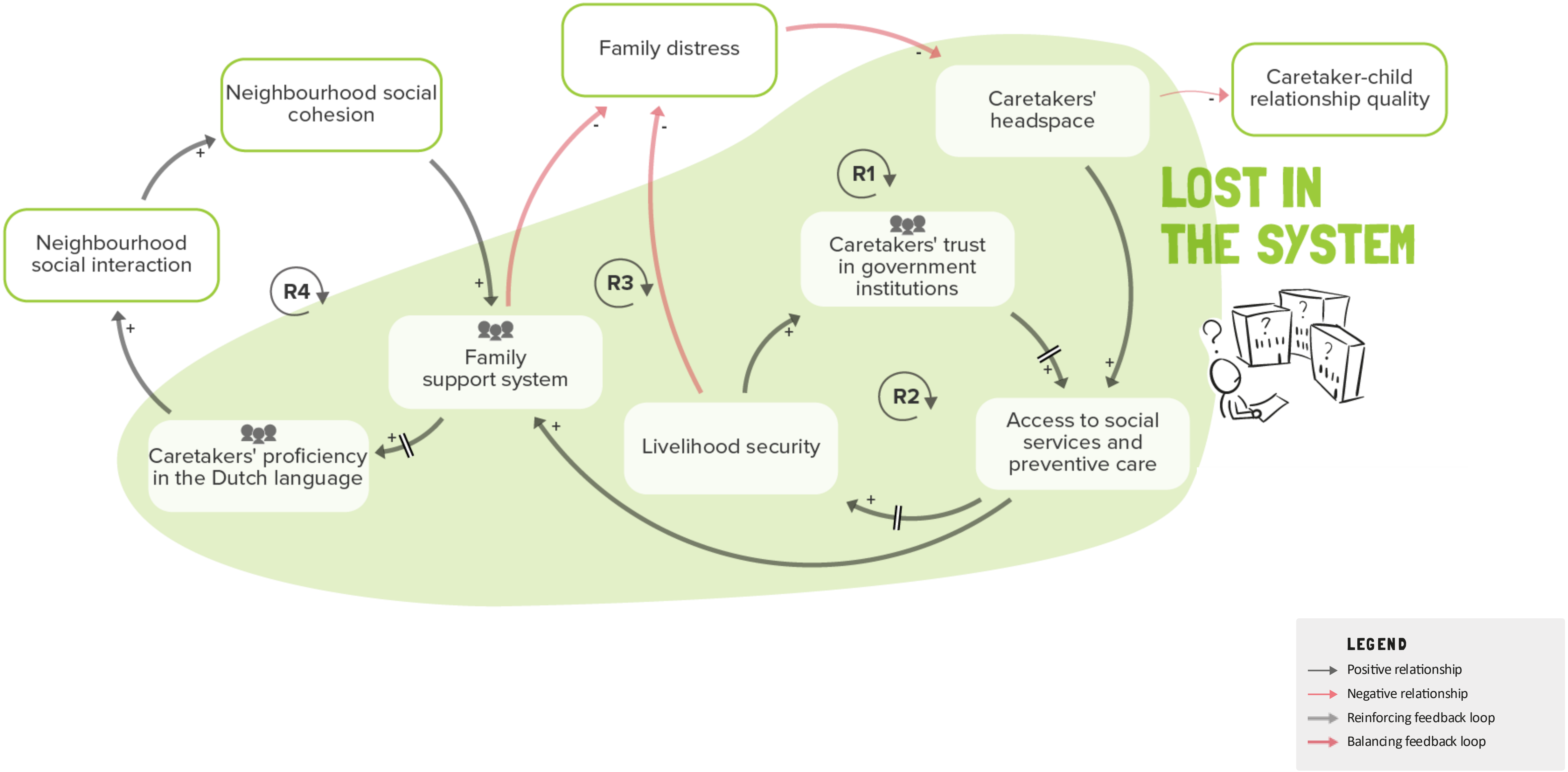
Family distress
Neighbourhood social cohesion
Caretakers' headspace
Caretaker-child relationship quality
Neighbourhood social interaction
R4
R1
R3
Caretakers' trust in government institutions
LOST IN THE SYSTEM
Family support system
R2
Livelihood security
Access to social services and preventive care
Caretakers' proficiency in the Dutch language
LEGEND
Positive relationship
Negative relationship
Reinforcing feedback loop
Balancing feedback loop

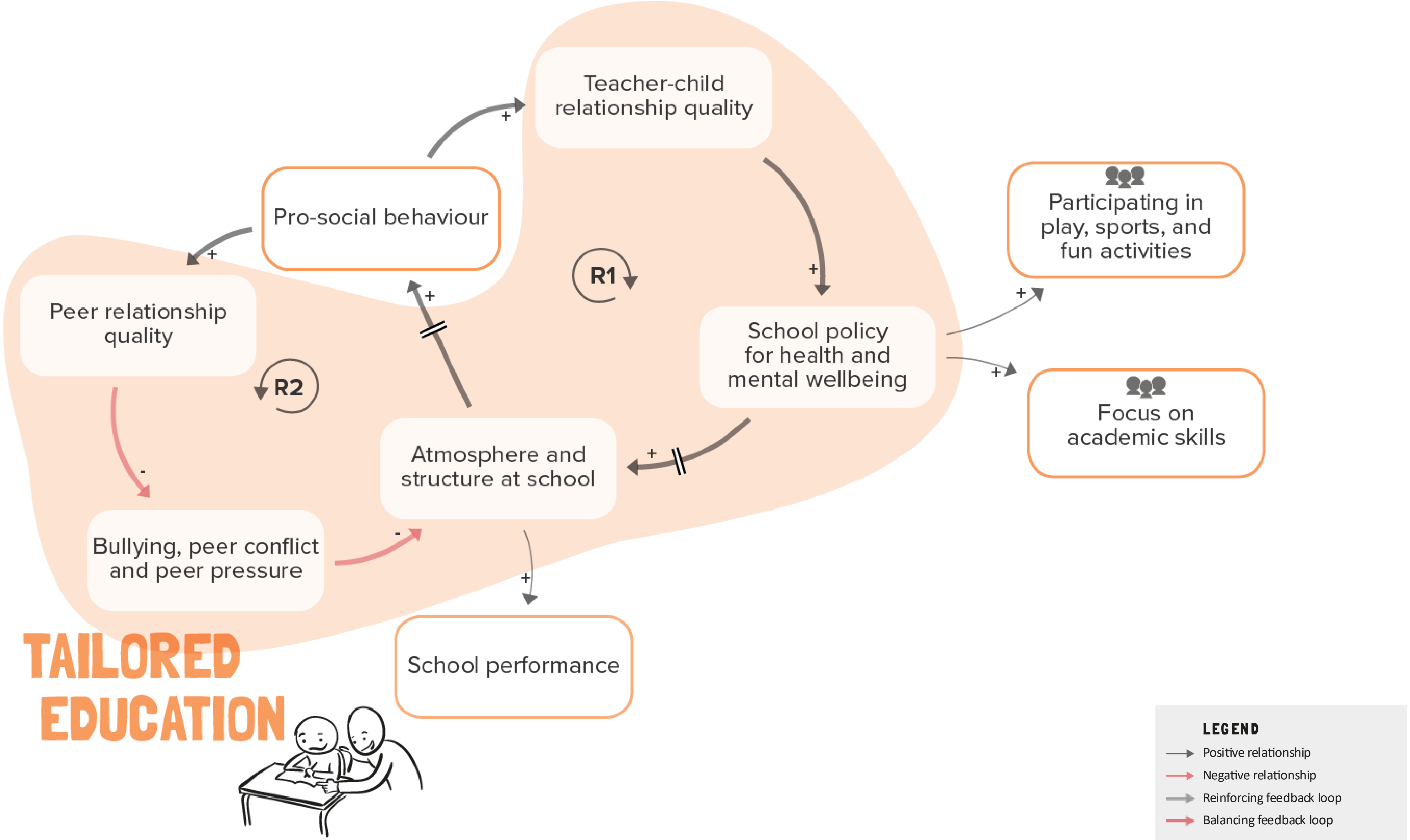

Teacher-child relationship quality
Pro-social behaviour
Peer relationship quality
R1
R2
School policy for health and mental wellbeing
Participating in play, sports, and fun activities
Focus on academic skills
Atmosphere and structure at school
Bullying, peer conflict and peer pressure
School performance
TAILORED EDUCATION
LEGEND
Positive relationship
Negative relationship
Reinforcing feedback loop
Balancing feedback loop
